# Federated Deep Learning Approaches for Detecting Ocular Diseases in Medical Imaging: A Systematic Review

**DOI:** 10.2174/0115734056400866250923175325

**Published:** 2025-10-02

**Authors:** Seema Gulati, Kalpna Guleria, Nitin Goyal, Ayush Dogra

**Affiliations:** 1 Chitkara University Institute of Engineering and Technology, Chitkara University, Punjab, India; £ Present Address: Graphic Era Hill University, Dehradun, India; 2 Department of Computer Science and Engineering, School of Engineering and Technology, Mahendergarh, Haryana, India

**Keywords:** Artificial intelligence, Deep learning, Federated learning, Ocular disease detection, Healthcare diagnostics, Data privacy

## Abstract

**Introduction:**

Artificial intelligence has significantly enhanced disease diagnosis in healthcare, particularly through Deep Learning (DL) and Federated Learning (FL) approaches. These technologies have shown promise in detecting ocular diseases using medical imaging while addressing challenges related to data privacy and security. FL enables collaborative learning without sharing sensitive medical data, making it an attractive solution for healthcare applications. This systematic review aims to analyze the advancements in AI-driven ocular disease detection, with a particular focus on FL-based approaches. The article evaluates the evolution, methodologies, challenges, and effectiveness of FL in enhancing diagnostic accuracy while ensuring data confidentiality.

**Methods:**

The systematic review followed the PRISMA (Preferred Reporting Items for Systematic Reviews and Meta-Analyses) framework to ensure transparency and reliability. Research articles published between 2017 and 2024 were identified using academic databases, including Web of Science, Scopus, IEEE Xplore, and PubMed. Studies focusing on DL and FL models for detecting ocular diseases were selected based on predefined inclusion and exclusion criteria. A comparative analysis of the methodologies, architectures, datasets, and performance metrics of different FL models has been presented.

**Results and Discussion:**

The findings indicated that FL preserves data privacy while achieving diagnostic performance comparable to traditional centralized AI models. Various FL models, including FedAvg and FedProx, have been implemented for ocular disease detection, with high accuracy and efficiency. However, challenges, such as data heterogeneity, communication efficiency, and model convergence, persist.

**Conclusion:**

FL represents a promising approach for ocular disease detection, balancing diagnostic accuracy with data privacy. Future research may focus on optimizing FL frameworks for improving scalability, communication efficiency, and integrating advanced privacy-preserving techniques.

## 1 INTRODUCTION

Artificial Intelligence (AI) has revolutionized the world around us through its practical applications in all walks of life, and the healthcare sector has benefited significantly from advancements in AI. The application of AI in the detection and early diagnosis of various diseases is a prominent example of how this technology is not only improving but also frequently perpetuating innovation in the healthcare industry [1]. Integrating AI in the healthcare industry through Machine Learning (ML) and Deep Learning (DL) algorithms has significantly enhanced the precision of disease diagnosis. These AI systems have shown considerable improvements in analyzing medical images, such as MRIs, X-rays, and CT scans, and have been instrumental in detecting various diseases with precision and, at times, outshine human experts [2-4]. The accuracy of prediction is pivotal for early detection, drastically improving detection accuracy in many cases. Additionally, the ability to process and analyze extensive medical data at a fast pace is invaluable, particularly in emergencies or in resource-limited settings. A prompt analysis accelerates disease diagnosis and helps healthcare professionals make smart decisions quickly for timely treatment [5, 6].

AI has significantly lessened the load of healthcare professionals by automating routine tasks, which helps them focus on more complex aspects of patient care. This reduces medical caregivers' work and improves patient care by providing a second opinion. Furthermore, the power of AI to exhibit exceptional performance in predictive analysis is revolutionizing the medical sector [7-9]. AI can predict individual disease risks by analyzing and mining vast datasets, including genetic and lifestyle factors, facilitating early treatment and personalized strategies. This approach has proved helpful in reshaping patient care, leading to better outcomes and more effective treatment. The continuous development and adaptation of AI systems ensure ongoing improvements in their performance, benchmarking a new era of AI in healthcare [10-12].

The term Federated Learning (FL) was first introduced in 2016 by Google, and it is a framework for the collaborative implementation of ML algorithms. At the time of introducing this new concept, global attention was on the misuse of sensitive user data. Federated learning emerged as a response to the security challenges posed by traditional AI models since it addresses key security concerns in AI by enabling model training across decentralized devices, which keeps sensitive data local and reduces privacy breach risks [13, 14]. Unlike centralized AI systems, which are vulnerable to large-scale cyberattacks, the decentralized nature of federated learning makes it harder for hackers to access vast datasets. It also assists in adhering to stringent data privacy regulations like GDPR, as data remains at its original location [15]. Additionally, federated learning is beneficial in industrial domains like healthcare and finance, allowing AI model development without compromising data confidentiality and enabling secure collaboration across organizations [16].

The Cambridge Analytica scandal sparked global debate about social media privacy, since the personal data of millions of users was breached to influence elections. Although the data was collected with consent, it was misused to benefit a particular political party [17]. Such incidents raise concerns about sharing personal information on any social media or online platform and the unauthorized tracking of user data [18-20]. Repeated data breaches by large companies impair public trust, prompting governments to enact stricter data protection laws. To address privacy concerns, Google introduced Federated Learning (FL), enabling collaborative ML/DL model training without transferring local data [21, 22].

The collaborative framework, termed federated learning, allows local sites to share their learning without sharing any of their original data, thereby maintaining the confidentiality of data at local sites. The foundation of FL is that the data is not shared at any level; only the learning is shared between the server and its client. This ensures that the local dataset at any participating site is never transported to another location. Instead, the learning is applied at that local site, and then the parameters after learning are sent to the server to update a global model. Since the data is not transferred, the threat of data leakage is reduced to a minimum [23, 24]. The approach also helps in building better predictive models since different organizations can participate in the learning process without divulging any of their sensitive data. This is quite fruitful in the healthcare industry, where different organizations can collaboratively train models to improve the efficacy of the trained models [25, 26].

This comprehensive review serves as a significant reference in understanding the current state and potential of AI, in particular, DL and FL, in the early detection and classification of ocular diseases. The main contributions of the article are given herewith:

• This systematic article presents an analysis of how artificial intelligence has revolutionized disease diagnostics in healthcare, with a primary focus on ocular diseases. It also presents the integration of federated learning in healthcare, which enables model training on decentralized devices while enhancing the privacy and security of sensitive user information.

• This systematic review article provides details of the evolution of federated learning, its architectures and applications, emphasizing the relevance of federated learning in managing distributed healthcare datasets that are geographically distributed or institutionally partitioned.

• A detailed analysis of state-of-the-art deep learning and federated learning models used for detecting and classifying ocular diseases like diabetic retinopathy, diabetic macular edema, cataract, and glaucoma is provided.

• The systematic review article presents an overview of various studies in the field, offering comparative insights into the applications and challenges of FL in ocular disease detection.

• This article highlights future research avenues in AI and FL for healthcare, emphasizing innovative solutions to enhance data privacy while addressing challenges like data heterogeneity, communication efficiency, and diagnostic accuracy.

• A critical review of privacy-preserving techniques, including differential privacy, homomorphic encryption, and secure aggregation, in federated learning models is presented in the article, with an emphasis on their significance in healthcare data management.

This comprehensive review article is organized into various sections to facilitate a clearer view and understanding for readers. The article probes into incorporating AI, especially DL and FL, in ocular disease detection. The second section outlines the research methodology (in accordance with PRISMA) employed in preparing the systematic review, including details of the research objectives, research questions, and selection criteria. The third section explores various reviews on federated learning, highlighting methodologies, applications, and limitations. The fourth section describes the fundamentals of federated learning, its workflows, architectures, and aggregation techniques. In the fifth section, models based on machine learning, deep learning, and federated learning for disease detection, with a focus on ocular disease detection, have been reviewed. Next, the sixth section presents a critical analysis of the various detection models identified in the fifth section and points out their limitations. The seventh section highlights the open issues and research challenges in FL, including data privacy, security, communication efficiency, and scalability. The article concludes in the eighth section by highlighting research gaps, proposing enhancements in federated learning for healthcare, and emphasizing its transformative potential in improving diagnostic accuracy, addressing data heterogeneity, and ensuring data privacy.

## 2 MATERIALS AND METHODS

### 2.1 Research Methodology

The systematic review for detecting ocular diseases by leveraging deep learning and federated learning adheres to the Preferred Reporting Items for Systematic Reviews and Meta-Analyses (PRISMA) guidelines for ensuring transparency, correctness, and reproducibility. The systematic review focuses on the advancements, methodologies, and challenges faced by employing FL for disease detection, especially ocular diseases. The research methodology includes several steps, as depicted in Fig. (**1**).

The steps followed for conducting the systematic review on leveraging deep learning (DL) and federated learning (FL) for the detection of ocular diseases are as follows:

1. Defining Research Questions and Objectives: It is vital to define clear research questions on the basis of the objectives, particularly in the context of applying deep learning and federated learning for the detection of ocular diseases. This focused approach will enhance the precision of the findings and contribute positively to advancements in patient care. The primary objectives of the review are to:

1. Thoroughly investigate existing reviews for federated learning in disease detection.

2. Highlight the challenges and limitations practically faced while implementing federated learning in disease detection.

3. Identification of the state-of-the-art deep learning and federated learning models used for disease detection, and in particular, ocular disease detection.

4. Determining the advantages of federated learning over traditional learning methods.

5. Assessing the role and effectiveness of federated learning in improving data privacy and enabling collaboration among healthcare institutions.

The research questions thus formed in order to accomplish the defined research objectives are given below:

RQ 1. What insights and trends can be derived from existing reviews on the application of federated learning in disease detection across various fields?

RQ 2. What are the primary challenges and limitations faced while implementing a federated learning framework for detecting ocular diseases?

RQ 3. What are the current state-of-the-art deep learning and federated learning models used in disease detection, particularly in ocular disease detection, and how do they compare in terms of performance?

RQ 4. How is federated learning advantageous compared to traditional centralized learning approaches, particularly regarding diagnostic accuracy, scalability, and privacy preservation?

RQ 5. How effective is federated learning in enhancing data privacy and facilitating collaboration among healthcare institutions for disease detection?

RQ 6. What challenges are associated with non-uniform and imbalanced datasets in federated learning frameworks for ocular disease detection, and how can they be addressed?

2. Outlining the methodology: It defines the framework for how the research will be conducted, ensuring transparency, rigor, and replicability. This step involves planning and documenting the procedures and criteria for every phase of the review.

3. Conducting a comprehensive literature review: A comprehensive search was conducted using academic databases, such as Web of Science, Scopus, IEEE Xplore, and PubMed. The keywords used in the search query were: Deep Learning, Neural Networks, Federated Learning, Ocular Diseases, Diabetic Eye Diseases, and Retinal Diseases.

4. Screening and selection of studies: It was conducted in two phases. In the first phase, the title and abstract were screened, followed by the second phase, where the full text of the articles was reviewed. Irrelevant or duplicate studies were excluded in the first phase. The selection process was documented using a PRISMA flow diagram given in Fig. (**2**). The inclusion and exclusion criteria for the screening of the articles are given below:

Inclusion Criteria:

The inclusion criteria are as follows:

• Articles with machine learning or deep learning models applied to ocular disease detection.

• Articles on federated learning applied to general disease detection or ocular disease detection.

• Articles published in English between 2017 and 2024.

• Peer-reviewed journal articles or conference papers in reputed journals and by well-established publishers.

• Research (implementation-based articles) reporting measurable outcomes, such as accuracy, sensitivity, specificity, privacy enhancements, dice similarity coefficient, or kappa score.

Exclusion Criteria:

The exclusion criteria are as follows:

• Articles not related to disease detection or healthcare applications.

• Articles published in languages other than English.

• Research published before the year 2017.

• Non-peer-reviewed literature (*e.g.*, preprints, editorials).

• Articles without an accessible full text.

• Research solely focused on traditional machine learning or deep learning components in diseases other than ocular diseases.

Research (implementation-based articles) that do not report measurable outcomes.

5. Extraction of relevant data: It involves the systematic collection of key information from the selected articles to address the research objectives and questions. The extracted data included:

a. Study Information: Authors, publication year, study location, and type of publication.

b. Methodology: Machine learning, deep learning, or federated learning models used, datasets, preprocessing techniques, and aggregation methods.

c. Outcomes: Diagnostic accuracy, sensitivity, specificity, privacy measures, and identified challenges or limitations.

d. Context: Application areas, healthcare settings, and collaborative frameworks.

6. Analyzing the data: both qualitative and quantitative analyses were conducted.

7. Disseminating the findings: The review findings were shared through a detailed report following PRISMA guidelines. Key insights, challenges, and opportunities have been clearly identified and are supported by relevant tables, figures, and statistical analyses. The report outlined the implications of the findings for researchers, practitioners, and policymakers, offering recommendations for integrating federated learning in healthcare. The results were prepared for publication in a peer-reviewed journal.

### 2.2 Related Work

This section examines various literature reviews on Federated Learning (FL), examining their methodology, datasets, performance, and key findings. The section also provides a summary of these contributions.

Li *et al*. [27] presented a comprehensive overview of FL, a privacy-preserving, decentralized ML approach. They addressed the challenges in data science and industrial engineering, such as data governance and data silos, and presented FL as an effective solution. The paper discussed the characteristic features of FL, which differentiate FL from traditional distributed systems. The authors highlighted key frameworks for implementing a distributed FL system, like TensorFlow Federated and Federated AI Technology Enabler, and described issues like communication overhead. They concluded by emphasizing the potential and need for continued research in FL. However, the paper does not detail the implementation-specific challenges and how to overcome them. It does not mention the different types of aggregation strategies and privacy techniques used in practice.

The systematic review by Crowson *et al*. [28] employed PRISMA guidelines and assessed studies using TRIPOD and PROBAST tools. The key findings of the article are that most studies focus on imaging results and binary classification *via* offline learning with a centralized topology, despite high compliance with TRIPOD guidelines. Nearly half (46.2%) exhibited a high risk of bias, and only a few studies used publicly available data. The review's limitations included its coverage of articles only up to 2020, challenges in comparative analysis due to the complexity of the content, and concerns about the reproducibility of the studies. The findings suggest that FL has been successfully applied in clinical domains, such as computer vision and oncology, but there are opportunities for improvement in terms of reproducibility and bias mitigation. The authors have not discussed the evolution of FL, its characteristics, and its categories. Additionally, the challenges in FL have not been taken into consideration in the review.

Yin *et al*., in 2021 [29], provided a detailed survey of the rapidly evolving field of privacy-preserving FL (PPFL), a scenario-based classification system. The article introduces FL and covers the related fundamentals, like the privacy-preserving mechanisms used in an FL system. In addition, the categorization of FL based on data partitioning criteria and communication architectures is also discussed. The article introduces various privacy techniques, metrics, and comparisons with related concepts. The survey investigates privacy leakage risks in FL and the current privacy-protection FL methods. However, the authors have not discussed the applications of FL in various domains and the aggregation methods used in FL scenarios.

Banabilah *et al*., in 2022 [30], presented an in-depth analysis of FL, a collaborative learning architecture initiated by Google and used in applications like Android's Gboard and Google Assistant. The article provides the fundamental concepts of FL, covering its technologies, architectures, and challenges with a particular focus on privacy concerns. The survey also highlights the application of FL across diverse fields and explores future trends and challenges in the practical deployment of FL. Although the privacy concerns are listed in the article, their solutions are not described in detail. Additionally, the efficient communication mechanisms are not discussed, and the aggregation strategies at the server are also missing.

Pfitzner *et al*., in 2021 [31], presented a systematic review of the literature on applying FL in the healthcare domain. The article reviewed the challenges of maintaining data privacy in medicine while using data for research and training ML models. It assessed the progress of FL in training algorithms, security protocols, privacy, and communication efficiency. The authors have emphasized the potential of FL in handling sensitive medical data and outlined challenges, including privacy-preserving hyperparameter optimization, entity resolution for vertically split data, and effective encryption methods. The survey does not discuss parameter aggregation methods or the various techniques used for updating the central or global model. Furthermore, communication-efficient strategies, which are crucial for an FL setting, have not been included in the survey.

Li *et al*., in 2023 [32], focused on the necessity of developing systems and infrastructures to support diverse FL algorithms, thereby correlating how systems like PyTorch and TensorFlow have propelled FL systems. The article emphasizes the significance of FL systems in addressing challenges related to effectiveness, efficiency, and privacy. The survey begins with the introduction of FL and later examines the key components of FL systems and the categorization of FL based on the data distribution, the model utilized, privacy mechanisms used, communication architectures followed, the federation scale, and the motivation behind it. However, the article focuses more on system design than on the practical application of FL and the implementation challenges faced. Additionally, privacy-preserving mechanisms and communication methods for efficient system convergence have not been thoroughly explored.

Kurupathi *et al*. (2020) [33] explored the integration of FL in privacy preservation for ML and DL models. The article provides details of various federated architectures like horizontal FL, vertical FL, and transfer FL. Additionally, the article elaborates on privacy-preserving mechanisms, including secure multiparty computation, homomorphic encryption, and differential privacy. It highlights the application of FL across industries for preserving privacy, but it has not discussed the applications where FL is currently used or potentially where it can be applied. The comprehensive survey refers to future research in the field, pointing out the potential and challenges of FL in privacy preservation. However, it has not delved into various aggregation methods instrumental in the convergence of the FL model. Additionally, communication-efficient methods also need to be discussed comprehensively.

Zhao *et al*., in 2023 [34], provided a detailed overview of communication challenges in FL systems and discussed and reviewed the latest research in this area. The focus is on three key aspects of communication: efficiency, environment, and resource allocation. The contributions of the paper are as follows: it organizes recent FL communication methods into a structured classification, compiles these methods into a comprehensive, easily understandable table, and identifies as well as proposes areas for future exploration and research within the realm of FL communication. The article focuses mainly on communication aspects, potentially overlooking other FL challenges like aggregation methods and secure communication methods. However, the background, such as the classification of FL based on different parameters and hyperparameters, remains unaddressed.

Vucinich *et al*., in 2023 [35], provided an analysis and summary of fundamental concepts, root causes, and various challenges related to fairness within the context of FL. The paper meticulously reviewed and discussed the latest techniques and methods that have been documented in recent scholarly literature, specifically aimed at addressing the challenges of ensuring fairness in FL systems. The primary focal point is that fairness and other challenges have not been studied comprehensively, and the other aspects, such as categorization, communication efficiency, practical applications, and implementations, have not been analyzed.

Huang *et al*. (2024) [36] worked on three aspects: generalization, robustness, and fairness of federated learning. The systematic literature review focuses on the foundational concepts, key challenges, developments in terms of methodology, and the benchmark methods in the domain. The authors also discuss open issues and future directions, such as in FL. However, the article relies on benchmarks, such as CIFAR-10 and MNIST; these traditional datasets are not representative of the real-world, complex, non-IID data. The article does not provide a detailed discussion of the architectural classification of FL based on datasets and devices.

Pei *et al*. (2024) [37] reviewed FL challenges in heterogeneous scenarios, focusing on the heterogeneity of devices, data, and models. The authors analysed how these heterogeneities impact the models’ training efficiency and accuracy, and categorised existing solutions, including the communication reduction techniques, adaptive model sizing, data preprocessing, model robustness methods, and improved frameworks. The review also discussed convergence theory for FL in non-IID settings and evaluated methods based on accuracy and communication costs.

The article by Chai *et al*. (2024) [38] presents a comprehensive review that includes key evaluation goals in federated learning (FL), focusing on utility, efficiency, and privacy. The authors discuss the model’s effectiveness and robustness, particularly under challenges, such as non-IID data and client heterogeneity. The efficiency covers both communication and the computations, which are critical due to resource constraints in FL environments. The article emphasizes the inherent trade-offs between these goals, where improving one often has a negative impact on the others. To address evaluation challenges, the authors introduce FedEval, an open-source platform designed to standardize and facilitate comprehensive FL assessments with realistic simulations and flexible metrics. While FedEval provides strong support for utility and efficiency assessments, the range of privacy and security attack simulations included is still limited and needs to be expanded to cover more diverse and sophisticated threats. The article lacks a standardised evaluation framework for heterogeneous federated learning, offers limited analysis of privacy-security trade-offs, and pays insufficient attention to large-scale real-world deployment challenges. It underexplores vertical federated learning, asynchronous and dynamic client participation, and concrete solutions for model heterogeneity. The article lacks concrete insights into practical deployment issues, and advanced methods, such as differential privacy, secure multi-party computation, or homomorphic encryption, are not analysed in depth.

Yuan *et al*. (2024) [39] in their comprehensive survey outlined the major advantages of decentralized federated learning, like elimination of the central server to save communication resources, enhancing privacy, and enabling more flexible peer-to-peer communication, in contrast to centralised federated learning. The authors discussed real-world applications across vehicles, healthcare, industrial IoT, social networks, and artificial intelligence. Although the article highlights the absence of centralised management as a major issue, it lacks any comprehensive frameworks or protocols for decentralised coordination, conflict resolution, or deadlock prevention, which remain largely unexplored. Additionally, the article lacks a detailed exploration of effective and scalable techniques in DFL for handling extreme heterogeneity among clients in various practical scenarios.

The comprehensive survey by Lu *et al*. (2024) [40] presents FL under realistic and challenging conditions of non-independent and identically distributed (non-IID) data. FL, as a decentralised machine learning approach, allows collaborative model training without sharing raw data, thereby preserving privacy, a critical concern in domains, such as healthcare, finance, and IoT. It explores the challenges of communication overhead, model convergence, performance bias, privacy, and security. It also reviews state-of-the-art techniques to mitigate these issues, including advanced aggregation methods, client selection strategies, adaptive communication protocols, and personalised FL. The article does not detail how to handle real-world challenges, such as scaling FL to multiple devices or addressing device limitations. Additionally, it does not fully address privacy and security issues that arise specifically due to non-IID data in federated learning.

Table **1** highlights the main aspects, key points, methodologies, discussed applications, shortcomings, and future research recommendations from each paper, providing a comprehensive overview of the current state and challenges in FL across different contexts.

### 2.3 Background of Federated Learning

FL is a novel technique that combines models from multiple client sites and servers to design efficient ML or DL models. All the data and computations are distributed across local sites; therefore, it is generally referred to as a decentralized and collaborative learning paradigm. The federated concept contrasts with the traditional centralized approach in terms of implementation, where multiple datasets are stationed at different organizations or geographically distant places. In the decentralized approach of federated learning, many sites participate in the learning process and have the databases saved on their local machines, whereas in the traditional centralized approach, the data is accumulated at one central location to form a single dataset, and then models are trained using this data. The learning models are applied at the sites where the data is held, as the data is distributed across different local sites. A central server is dedicated to the coordination of the learning process at a global level. The dedicated central server communicates the parameters and weights of the global model to be used for learning at the client end [41].

#### 2.3.1 How Federated Learning Works

The central server acts as the controller and administrator of the whole process of learning. The process begins with the server applying training using a DL model (usually a pre-trained model) at its location, called the Global Model. The weights and parameters of the central or global model are then transported to the participating or selected clients in an encrypted form. On receiving the weights and parameters of the global model, training is done locally for the clients using the same algorithm and parameters with their local dataset [42, 43].

The client devices that undergo local training using the global weights optimize the weights based on their local training, and the model developed is referred to as the local model. After all clients have finished their training, the locally updated model weights are sent to the server. The server will aggregate the weights after receiving updated weights from the participating clients to form a new global model, which is an aggregation of the updated weights of the local models. The process is iteratively repeated to improve the efficacy of the global model, and the iterations continue until the model stabilizes and shows no further improvement in terms of prediction accuracy and precision.

The conceptual difference between FL and traditional centralized learning is the accumulation of datasets in a single place. The traditional method involves the dataset being transported or collected on one central server, and then ML or DL is applied. In contrast, FL does not pool the data; in fact, the data is never touched in terms of relocation, and the model is applied at the same place where the data is produced and stored. This difference is represented in a diagrammatic form in Fig. (**3a**), which illustrates centralized learning, and Fig. (**3b**), which illustrates the FL framework [44].

FL was introduced to address the privacy issues of users and prevent the misuse of personal data. The basic principle of FL is that the information stored in the system is private to the site storing the dataset, and none of the data leaves the system ever, and stays protected in the container site. Instead, the training is done at the device level, and only the weights and parameters required are shipped. The motivation behind this is to protect sensitive user data without compromising the efficiency of training. FL opens gateways for many DL applications, which will enable training on real-world data and designing application-specific models without any privacy breach [34].

#### 2.3.2 Characteristic Features of a Decentralized, Collaborative Federated Learning Framework

FL is strongly backed by its characteristic features, which enable the practical feasibility of an FL-based framework. These fundamental characteristics that distinguish it from traditional centralized AI systems are outlined and described below:

i. *Data Diversity and System Heterogeneity:* If we compare a simple distributed system, which is employed to only alleviate system performance by increasing the degree of parallelism, the data used will be independent and distributed identically among nodes. In contrast, the data used in an FL setup is usually non-independent and not identically distributed since the raw sets of data are inaccessible to the FL server [45, 46]. Therefore, the distribution of data in such an environment is usually imbalanced, and the probability distribution of the training samples also varies significantly. Furthermore, the performance of the system, which is dependent on the feature extraction from the local data, fluctuates in accordance with the stored labels [47, 48].

ii. *Ensures Privacy and Encourages Resource Sharing:* Privacy protection in FL refers to the methods and techniques employed for the assurance of the security and confidentiality of sensitive data during the training process. In FL, the training dataset is scattered across multiple client devices, and the data remains locally stored on their machines and never leaves the place. Instead of sharing the raw data, FL shares only the gradients or updates of the central model, which are later aggregated according to the selected criteria, for building a global model without exposing individual data points. This process empowers the data owners to exercise control over their sensitive and confidential information, while still collaborating on the central model's improvement. Privacy protection mechanisms in FL may include techniques like differential privacy, secure aggregation protocols, encryption methods, and FL with fully homomorphic encryption (FHE) [49, 50]. The objective is to strike a balance between collaborative learning and data privacy, enabling multiple parties to collaborate on building a robust global model while keeping their data safe from exposure or misuse. Resource sharing in FL refers to the practice of distributing the training workload across multiple devices or nodes, thereby leveraging the computational power and data storage capabilities of each participating entity. In FL, each device or node performs local training on its respective data, and only the model updates are exchanged during the aggregation process [51]. Resource sharing offers several benefits in FL, such as reducing the burden on individual devices, enabling more efficient and scalable training, and utilizing the collective computational resources available across the network. By sharing resources, FL enables the participation of a wide range of devices, including edge devices, mobile phones, IoT devices, and servers, in collaborative ML tasks, fostering a distributed and collaborative learning environment. In simpler terms, privacy protection ensures that individual data remains secure and confidential throughout the FL process, while resource sharing enables efficient and distributed training across a network of devices or servers [52].

These two aspects together form the foundation of FL, enabling privacy-preserving and collaborative ML at scale.

iii. *Iterative Learning Process of a Federated Deep Learning Model:* The concept of FL involves building a central global model with good prediction accuracy; in other words, it should attain high efficacy in predicting outputs. The federated method is dependent on an iterative learning process, which involves a series of communications between the server and clients, referred to as FL rounds. Each communication round consists of the transmission of the present central model state to the participating clients, and then training is carried out using this state locally on the dataset stored at the client's machine [53, 54]. After training, the clients will again update the model locally and again transmit the amendments made to the server, which further incorporates all the amendments of the participating clients using an aggregation strategy. Thus, after every communication round, the clients collectively upgrade the central global model, building a new state of the global model. The process halts after several iterations when the model stabilizes, and further updates are not able to improve the model significantly. Therefore, the complete iterative process, as shown in Fig. (**4**), shall terminate when the efficiency of the current model no longer increases and becomes stable [55].

iv. *Decentralization of Technology:* As discussed earlier, in a centralized approach, the dataset is pooled at a central device, which becomes the server. The central server is responsible for the training in a centralized and distributed approach to ML. The data is divided among other nodes by the server, and these nodes then implement the learning model, all of which are controlled by the server. The FL is decentralized in the sense that, unlike a centralized framework, a pool of data is never formed in an FL framework. The designated server is not only responsible for initiating the learning process but also for aggregating the client models to build an efficient global model. The server acts as a coordinator for the clients, whose job is to form a global model based on the clients' local learning [56, 57]. Therefore, the learning process for the clients is completely independent, and the server does not control the client's learning process. Both the client learning process and the data are out of the jurisdiction of the server. Again, this makes it an even more challenging task to implement the FL framework [58, 59].

#### 2.3.3 Classification of Federated Learning Architectures

The architecture of Federated Learning (FL) is broadly categorized based on two key attributes: first, the type of data being used, and second, the type of participants involved in the learning process. This classification helps in understanding how FL is applied in different scenarios and the nature of the participants that are part of the federated network [25, 60]. The classification of FL is diagrammatically shown in Fig. (**5**).

Further, FL can be classified into two categories based on the participating devices and the three categories based on the dataset used [61].

The type of participants is a characteristic that distinguishes between two different types of FL architectures, which are explained below:

i. *Cross-device Federated Learning*: This type involves a large number of devices (potentially millions), each holding a small amount of data, as depicted in Fig. (**6a**). Common examples include smartphones and wearable devices like smartwatches. Each device participates in the learning process by contributing data from its local environment, making it a highly distributed form of FL. Examples of devices include smart mobile phones and wearable devices, such as watches [62].

ii. *Cross-silo Federated Learning:* Contrastively, cross-silo FL typically includes a smaller number of participants, as shown in Fig. (**6b**), but each holds a larger quantity of data. This form of FL is common in institutional settings like hospitals, banks, or universities, where large databases are collaboratively used for training models without compromising the confidentiality and integrity of the data [63, 64].

Federated Learning (FL) is an advanced approach to machine learning where both the data and model training are distributed across multiple devices or locations. Depending on the nature of the data and the specific requirements of the application, this methodology can be implemented in various ways and is adaptable. The categorization of FL into horizontal, vertical, and federated transfer learning represents distinct strategies for employing this technology.

i. Horizontal Federated Learning: This type of FL is employed when different data sources (such as various hospitals) have similar features, but the data samples are different, as shown in Fig. (**7a**). In this scenario, the objective is to learn a model collaboratively across these sources located at different sites, while ensuring that the data remains in its original location. For instance, this may involve multiple hospitals where each hospital scans chest X-ray images for COVID-19 detection. Despite using different scanning equipment, their goal is to identify the same features (signs of COVID-19) in the images. Horizontal FL allows these hospitals to collaboratively learn from their collective data without sharing the actual data, thus preserving privacy [65, 66].

ii. Vertical Federated Learning: This FL approach, as shown in the diagrammatic representation in Fig. (**7b**), is useful when different entities have overlapping data samples but seek to identify distinct features within this data. In such cases, each entity holds a unique set of features about the same set of samples. A practical example could be a medical organization where the radiology and pharmacy departments of a hospital have access to the same patient data. The radiology department focuses on imaging data, while the pharmacy department will be interested in medication and prescription data. Vertical FL will allow them to train models on their specific features according to their requirements without needing to access each other's data, maintaining data privacy and integrity [25, 67].

iii. Federated Transfer Learning: This form of FL, diagrammatically presented in Fig. (**7c**), is designed for situations where the datasets involved are significantly different in both samples and features, with minimum or nothing in common. It is particularly useful for developing specialized, application-specific models where data cannot be easily pooled or shared due to privacy concerns of sensitive user data or logistical challenges. In federated transfer learning, the learned parameters from one dataset (or domain) are adapted or transferred to a different but related dataset (or domain). Thus, enabling the development of robust models despite the lack of large, centralized datasets. This approach is instrumental in applications where data is scarce or highly specific to certain conditions [68, 69].

In summary, these three categories of FL offer flexible and privacy-conscious solutions for a wide range of data scenarios in machine learning. These are particularly useful in fields like healthcare, where data sensitivity is a prime concern [70].

#### 2.3.4 Techniques for Aggregating Model Parameters for the Central Model

The conventional learning approach collects data at a central server, which applies learning techniques to the pooled data for prediction and classification tasks. On the contrary, in an FL setup, the data is localized at the nodes that produce the data, and a data bank is not created at the server; the learning models are shared between the clients and the server to perform training locally on the client side [71]. The local training at the client site is implemented for maintaining the privacy of the data, and the data used in the training process resides at the local location only. In the FL paradigm, the term ‘exchange of models’ is often used synonymously to convey model communication; however, it does not elaborate on how the messages are exchanged. Two main approaches to message communication in an FL scenario are exchanging gradients and exchanging parameters.

*Commuting Gradients*: As the term suggests, the gradients computed locally at the client side are commuted by the client to the server, rather than exchanging the entire model. All clients compute the gradients locally while implementing the model using data residing on their site. These gradients are then sent to the server, which is responsible for updating model parameters in such a manner that the loss function is always maintained at a minimum. The exchange of gradients lowers communication costs while preserving privacy.

*Commuting Weights*: The weights here refer to the values allocated to the linkages of the neural networks used in DL models. The links are between the layers of neurons, and the output of one layer serves as input to the following layer. The weights of these links are adjusted to minimize the deviation between the expected and obtained results. The weights are passed back and forth between the server and its clients to upgrade the learning models and improve the predictions made by the model. This technique is favourable in the FL paradigm since it lessens the cost of message communication when compared with full model transmission.

a. *Federated Averaging (FedAvg):* The algorithm was first proposed by the company Google, and this algorithm transfers the parameters of the learning model instead of the complete model. Therefore, it is one of the communication-efficient strategies for FL. The data is securely kept by the clients, and a designated server at a central location is responsible for correspondence. This server will share the global parameters and gather the locally updated values of these parameters from the client nodes. The algorithm works well with data distributed identically, and the performance of the model deteriorates when the averaging is applied in a non-IID scenario [72].

Initially, the algorithm starts by arbitrarily assigning weights to the global model. In a single loop of averaging, the server selects the client that will participate in the process and then distributes weight to these clients. The clients will then work on the local data and update these weights at their end according to the learning and send them to the server. The server will then take the average of these locally revised weights and amend the global weights, incorporating the average. There is a trade-off between the prediction accuracy and communication based on the number of epochs used for training and the learning rate of the model.

b. *Federated Stochastic Gradient Descent (FedSGD):* Normally, SGD works on mini-batches, which are small partitions of the complete database, but in a federated scenario, the database is already distributed among the client nodes and thus partitioned. In the FedSGD algorithm, the global model is commuted to the local clients, which then compute the gradients locally using their resident data partition. The gradients by the clients are then shipped to the server, and at the server end, the locally obtained gradients are aggregated. The aggregation is done based on the ratio of the records that the clients hold.

c. *Federated Learning with Dynamic Regularization (FedDyn):* In this strategy of aggregating the FL clients, the server arbitrarily selects a fragment of the clients that participate in the current round of learning. The computations are generally made on the client side in order to reduce the cost of transporting. Regularization is done at the local and global levels to reduce the empirical losses. The task of the regularizer is to penalize the loss function to improve generalization. The loss at the local client device level is much less in comparison to the losses at the global level. In the algorithm, the participating nodes are allocated a regularizer, and the nodes of the current round update the regularizer regularly to check the compliance of local loss with the loss at a global level. The regularization is dynamic, ensuring the convergence of local losses to global losses.

d. *FedPROX:* FedProx is considered an upgrade of the FedAvg strategy of aggregation. The algorithm was launched to cater to the diversification of the federated networks. The algorithm is modified in terms of parameters with only slight changes to the algorithm. The FedProx can be considered a mutant of the original FedAvg algorithm, and the proximal operator is added to the algorithm instead of the local gradient descent. The algorithm caters not only to diversification but also allows different ratios of the task to be performed at the client nodes, and a proximal term is used to stabilize the client updates. A subset of the clients is chosen from the set of clients, and the chosen lot will participate in the current round of FL. The proximal term at each client is negatively rewarded if there is a significant deviation from the server model. The server should aggregate the weights of only those clients that show less deviation [73].

### 2.4 Literature Review on the Detection of Diseases with Deep Learning and Federated Learning Models

This section offers an overview and analysis of existing research related to the detection of diseases, particularly emphasizing the application of DL and FL models in this field.

#### 2.4.1 Deep Learning Models for the Detection of Ocular Diseases

This section delivers a comprehensive review of literature focusing on the application of DL models in the detection and analysis of ocular diseases.

i. Automated detection and grading of diabetic macular edema from digital colour fundus images

Rekhi *et al*., in 2017 [74], presented an approach to detect DME (Diabetic Macular Edema), which is robust in nature. Detection is based on the fundus image dataset obtained from open-source databases: MESSIDOR and DIARETDB1. The detection of the macula is based on the morphological features. The segmentation is done using the support vector machine (SVM) ML algorithm. The proposed methodology consists of four steps: normalization of the fundus images, exudate segmentation using anisotropic diffusion on green channel images, detection of the macula by applying histogram equalization to grey-scale images, and grading the disease based on exudate location. On the DIARETDB1 dataset, the algorithm achieved an accuracy of 95.45% for detecting normal fundus images, 92.11% accuracy for severe cases of DME, and 87.5% accuracy for moderate DME cases. When tested on the MESSIDOR dataset, the method achieved an accuracy of 92.72% for normal images, 90% accuracy for severe DME cases, and 88.89% for moderate DME cases.

ii. An exudate detection method for the diagnosis of the risk of diabetic macular edema in retinal images using feature-based and supervised classification

Marin *et al*., in 2018 [75], presented a system to detect the risk of DME using the fundus images. The presence of exudates helps in the timely detection of the disease. The authors employed feature-based techniques and supervised classification for detecting DME in the fundus images. The proposed work was evaluated on a subset of 1058 coloured fundus images, which were in correspondence to 529 patients with diabetes. Each patient had two macula-centered retinographies, one for each eye. The set of training and testing images was obtained from the MESSIDOR (Methods to Evaluate Sensitivity to Diabetic Retinopathy) public database. The specificity score was around 70%, and the sensitivity score was approximately 90%. These values are promising, and even better scores can be obtained with more training.

iii. A deep learning ensemble approach for diabetic retinopathy detection

Qummar *et al.* (2019) [76] focused on the classification of Diabetic Retinopathy (DR) into five stages: normal, mild, moderate, severe, and proliferative. The learning method employed was a deep CNN. Five models of CNN were utilized, *i.e.*, Dense121, Dense 169, Resnet50, Xception, and InceptionV3, to extract the features that improve the accuracy of classifying the various stages of DR. The dataset was curated from the open internet resource, Kaggle, and it consisted of 35126 images. These images were coloured fundus images of 5 categories according to the severity of the disease. The dataset was a compilation of 35126 colour fundus images. The set of images belonged to five classes based on the severity of diabetic retinopathy (DR). The five stages of DR progressed from mild to advanced in the order of: no DR or normal, mild, moderate, severe, and proliferative DR. An ensemble model, combining other ML models, was used to improve the prediction of the multi-class classification task. It is a combination of 5 CNN models: Dense-169, Dense-121, Xception, Inceptionv3, and Resnet50. The average accuracy achieved was 80.8%, recall was 51.5%, specificity was 86.7%, precision was 63.8%, and the F1-score was 53.7%.

iv. Automated diabetic retinopathy detection based on a binocular siamese-like convolutional neural network

An approach was devised by Zeng *et al*. in 2019 [77] for detecting referable diabetic retinopathy (RDR) automatically using fundus photos. A siamese-like structure was designed with deep CNNs for classification. The image dataset used in the work was obtained from the open-source Kaggle repository. The dataset used in the work is a high-resolution image set called DR competition provided by EyePACS (Eye Picture Archive Communication System). The dataset contained 35126 high-resolution fundus photographs that have been labelled by trained clinicians on a 5-point scale, starting from 0 and going up to 4. The authors demonstrated that the proposed approach achieved an AUC score of 0.951, a sensitivity score of 82.2%, a specificity score of 70.7%, and a kappa score of 0.829.

v. An adoptive threshold-based multi-level deep convolutional neural network for glaucoma eye disease detection and classification

Aamir *et al.* (2020) [78] presented a DL approach to detecting and classifying glaucoma disease into different stages, *i.e.*, early, advanced, and moderate. The authors introduced a multi-level deep convolutional neural network (ML-DCNN) that operates in two phases. In the first phase, a detection net was used for detecting glaucoma, and then in the second phase, the classification net was used for classifying images into advanced, moderate, and early glaucoma. The dataset consisted of 1,338 retinal fundus images that were collected from a local hospital with different stages of glaucoma. The proposed ML-DCNN model achieved 99.39% accuracy, 97.04% sensitivity, 98.99% specificity, and 98.2% precision. The authors demonstrated a superior performance in glaucoma detection.

vi. Support vector machine-based method for automatic detection of diabetic eye disease using thermal images

Selvathi *et al.* (2019) [79] devised an approach to automatically detect diabetic eye diseases (DEDs) from thermographic images of the eye. The proposed technique used thermal variations in the structure of the eye for the identification of abnormalities reflecting the presence of DEDs. The thermographic images were pre-processed, and then texture features were extracted from grayscale images using the Gray Level Co-occurrence Matrix (GLCM). The extracted features were then classified using an SVM classifier. A 5-fold cross-validation technique was applied to enhance the generalizability of the model. The dataset containing thermal images was a curation of 283 images, of which 149 suffered from diabetes and 134 were healthy individuals, and this dataset was taken from the Indira Gandhi Centre for Atomic Research (IGCAR), Kalpakkam. The binary classification of the healthy and diseased eye achieved an accuracy score of 86.22%, a specificity of 79.17%, and a sensitivity of 94.07%.

vii. Multi-label classification of retinal lesions in diabetic retinopathy for automatic analysis of fundus fluorescein angiography based on deep learning

Pan *et al.* (2020) [80] worked on fundus fluorescein angiography (FFA) images for the automatic detection and multi-label classification of DR. The dataset containing 4067 images used by the authors was collected from an Eye Center at an affiliated hospital of Zhejiang University School of Medicine. The proposed method involved the identification of 4 types of lesions, *i.e.*, microaneurysms, non-perfusion regions (NP), laser scars, and leakages for the detection of DR. The methodology adopted by the researchers included using 3 CNN-based models for training- DenseNet, ResNet50, and VGG16. Out of the three, DenseNet performed the best, with AUCs of 0.8703, 0.9435, 0.9647, and 0.9653 for detecting NP, microaneurysms, leakages, and laser scars, respectively.

viii. JointRCNN: A region-based convolutional neural network for optic disc and cup segmentation

Jiang *et al*., in 2020 [81], proposed a DL-based approach to detect glaucoma eye disease by jointly segmenting the optic disc and cup. The authors proposed a region-based, end-to-end CNN, incorporating atrous convolution to enhance feature extraction. These regions further help in detecting the presence of glaucoma disease. The authors introduced a disc proposal network (DPN) and a Cup Proposal Network (CPN) to generate bounding box proposals for the optic disc and cup, using a disc attention module to connect the two networks, and computed the vertical cup-to-disc ratio for glaucoma detection. The dataset used in this case study was obtained from the Singapore Chinese Eye Study (SCES) dataset and the Online Retinal Fundus Image Database for Glaucoma Analysis (ORIGA), comprising 650 and 1,676 images, respectively. The results are based on an AUC value of 0.901 for SCES data and 0.854 for ORIGA data, representing an improvement over all current published works in joint segmentation.

ix. A deep learning interpretable classifier for diabetic retinopathy disease grading

de la Torre *et al*. (2020) [82] presented a deep learning model for classifying diabetic retinopathy into various levels or grades based on the complications developed in the eye. The approach justified the results obtained by classifying the image, allocating a score to each point. The allocated score served as an indicator of the benefit of that pixel in the conclusive classification. These scores were calculated based on a score propagation model, which operates on each pixel for every neuron in the network. The images used in the study were from the dataset uploaded on Kaggle by the EyePACS platform for the detection of DR, with 75,650 images used to train the machine and another 10,000 images for testing the model. Even though the dataset was sufficiently large, the test accuracy of the approach was found to be 0.857 (85.7%) and 0.910 (91.0%) when tested on the EyePACS dataset and MESSIDOR dataset, respectively.

x. Automated Detection of Mild and Multi-class Diabetic Eye Diseases using Deep Learning

Sarki *et al*. (2020) [83] worked on mild and multi-class DEDs using pre-trained CNNs. Two pre-trained CNNs, *i.e.*, VCG16 and Inception V3, were applied to the datasets using Anaconda, Keras, Python, and TensorFlow platforms. The training data consisted of 1748 images from the MESSIDOR subset, 101 images from the Drishti-GS dataset, and 100 cataract images from GitHub’s Retina dataset, all of which were used in the study. Initially, for mild DED classification, VCG16 achieved an accuracy of 82.42%, while InceptionV3 achieved an accuracy of 78.52%. After fine-tuning and optimization, the accuracy improved to 85.94% for VCG16, but remained the same for InceptionV3. Additionally, for the multi-class classification of DEDs, the accuracy improved from 84.88% to 88.3% for VCG16, and for Inception V3, it increased from 79% to 81%.

xi. Integrating Deep Learning with Electronic Health Records for Early Glaucoma Detection: A Multi-dimensional Machine Learning Approach

Karimi *et al*. (2024) [84] focused on integrating deep learning (DL) models with electronic health records (EHRs) to enhance early glaucoma detection. Three machine learning models, namely random forest, gradient boosting, and sequential, were utilized to analyze EHR data. A multidimensional approach that incorporates clinical variables, such as intraocular pressure, BMI, demographic information, and lifestyle factors like tobacco and alcohol use, was employed. The dataset comprised de-identified EHR data from 1,652 participants, with an equal split between 826 patients with glaucoma and 826 control subjects. Random Forest outperformed other models with an accuracy of 67.5% and a ROC AUC of 0.67, highlighting its robustness in handling complex datasets.

xii. Fundus photograph-based cataract evaluation network using deep learning

Gao *et al*. (2024) [85] developed a Dual-Stream Cataract Evaluation Network (DCEN) to classify cataract types and grade severity using fundus photographs. The study utilized 1,340 color fundus photographs collected from 875 participants, aged 50 to 91 years, as part of the Beijing Eye Study 2011. The ResNet50 model was used for type classification and severity grading. The performance for classification was measured to be 97.62% accuracy, 94.01% F1-Score, and 0.8618 Kappa score.

xiii. Deep Learning-based Classification of Eye Diseases using a Convolutional Neural Network for OCT Images

Elkholy *et al*., in 2024 [86], applied a CNN-based approach for detecting three retinal diseases, Diabetic Macular Edema (DME), Choroidal Neovascular Membranes (CNM), and Age-related Macular Degeneration (AMD), along with the normal condition. The research utilized a publicly available dataset comprising a total of 35,468 OCT images, which were equally partitioned into four classes of disease. After adjusting the contrast and brightness, a Gaussian blur was applied to reduce noise. The authors leveraged the transfer learning approach with the VGG16 DL architecture. The model initially achieved 94% accuracy, and after fine-tuning, it improved to approximately 97% accuracy on the test dataset. However, the potential challenges of imbalanced data and significantly high computational requirements have not been addressed.

xiv. Deep learning-based CNN for multiclassification of ocular diseases using transfer learning

Deepak *et al*., in 2024 [87], optimized CNNS for multi-class classification of ocular diseases- glaucoma and cataract using transfer learning. The authors compared the implementation results of three pre-trained DL models, namely EfficientNetB0, DarkNet53, and SqueezeNet. They hyper-tuned parameters by varying batch sizes and trying different optimizers. The best-performing DL model on the dataset was DarkNet53 with 99.4% accuracy, which used the ODIR dataset from Kaggle, featuring 5,000 ocular fundus images. The DL model DarkNet53 is a complex model with 53 layers, and these may cause challenges in deployment on resource-constrained devices. The authors investigated the effects of batch size and optimizer type; however, other hyperparameters, such as learning rate schedules, dropout rates, and weight initialization methods, have not been explored.

Table **2** summarizes the machine learning and deep learning models used in detecting ocular diseases, giving the methodology, dataset, and key performance metrics.

#### 2.4.2 Detection of Eye Diseases using Federated Learning

This section presents a literature review focused on the detection of eye diseases utilizing FL techniques, offering insights into the current research and advancements in this specialized area of study.

i. Federated Learning for Diabetic Retinopathy Detection in a Multi-center Fundus Screening

Matta *et al.* (2023) [88] proposed a collaborative model for classifying images into normal and RDR categories. The term 'referable' has been used in the context of a condition when referring the patient to an eye doctor is necessary. The referable stages considered were moderate retinopathy, also known as severe non-proliferative diabetic retinopathy, and proliferative retinopathy. The key DL algorithm used was the EfficientNet-B5 algorithm for both server and client modules. Both weighted and unweighted averaging methods were used as the aggregation strategies for the client-server setup. The multi-centre collaborative model was developed using the OPHDIAT dataset, which contains fundus images in RGB colours. Certified ophthalmologists labelled these images in accordance with the ICDRS. A total of 697,275 images were used for the fabrication of the FL setup. From these, 641,917 images were utilized for training, and a subset of 21,054 images was utilized as the testing set. The performance metric used for comparing the fabricated FL model and the centralized DL methods was the area under the curve (AUC). The performance achieved by the emulated federated models was similar and, thus, comparable to that of the centralized model, which is a small trade-off for the privacy of the sensitive data of the patients.

ii. Privacy-Preserving Federated Learning with Domain Adaptation for Multi-Disease Ocular Disease Recognition

Tang *et al*. (2023) [89] proposed an FL model for multiclass classification of 7 eye diseases, namely, glaucoma, diabetic retinopathy (DR), myopia, age-related macular degeneration (AMD), cataract, hypertension, and others. To ensure data privacy, a two-phase mechanism involving Gaussian randomization was applied. The approach incorporated domain confusion and a multi-expert learning methodology to safeguard data privacy while addressing the domain gap issue. The data was divided into two types: monocular data, which are images from one of the eyes of a person, and binocular data, which are the images taken from both eyes of the person. If monocular data were used, only one feature was obtained using a feature extractor. For the binocular data, there were two feature maps for both eyes of the person. In the case of 2 feature maps, concatenation was done to obtain a single feature map. The OIAODIR dataset, compiled by Shanggong Medical Technology Co., Ltd. from multiple hospitals in China, comprised anonymized data from 5,000 patients, including age, color fundus photographs of both eyes of a person, and diagnostic keywords provided by doctors. This dataset represented a real-world collection of patient data compiled by Shanggong Medical Technology Co., Ltd. The dataset used contained a total of 10,000 images labelled by ophthalmologists with two or more years of experience. Metrics for evaluation of the performance of the proposed FL setup were comprised of the F1-score and the area under the curve (AUC). The ResNet50 DL architecture was used as the foundational model. The highest AUC was 0.7946 for off-site and 0.7684 for on-site, while the F1-score was 0.8500 for off-site and 0.8512 for on-site. The ablation study revealed that the experiment enhanced cross-domain classification performance while preserving the confidentiality of sensitive patient data.

iii. Federated Transfer Learning for Diabetic Retinopathy Detection Using CNN Architectures

Nasajpour *et al*., in 2022 [90], focused on the detection as well as grading of DR using a collaborative FL model [90]. The authors implemented two different aggregation strategies: FedAvg and FedProx, and compared their performance with that of a centralized DL system. The AlexNet model was used for training the central model and the local models. The dataset used was curated from 5 different public domain datasets: EyePACS, MESSIDOR, University of Auckland (UoA), APTOS, and IDRID. The curated dataset included approximately 3,000 colored fundus images. The metrics used for evaluation were accuracy, sensitivity, precision, and specificity. The authors found that the accuracy of the federated models was comparable to that of the standard model, *i.e.*, 90.07%, for the federated model with the FedAvg aggregation strategy, and the FedProx aggregation provided 85.81% accuracy, while 92.19% accuracy was achieved by the standard model.

iv. DRFL: Federated Learning in Diabetic Retinopathy Grading Using Fundus Images

Mohan *et al*., in 2023 [91], proposed a CNN-based FL that captured the characteristics of fundus images at local, global, and intermediate levels. The model implemented the FedAvg aggregation method and the median cross-entropy loss. A CNN-based algorithm was used for the training of the local and global models. The implementation simulated 5 clients, each representing a hospital and a central server for coordination and development of a central model. The central extracted multi-scale features for the identification of small lesions in the images. The dataset was a set of pre-processed fundus images available in public open sources and a locally procured database at Silchar Medical College and Hospital. It included 5000 images from open sources and a locally acquired dataset. From these 5000 images, 4000 were used for testing, and 1000 were used for training. The overall accuracy score achieved was 98.6%, the specificity obtained was 99.3%, while the precision and F1-score were approximately 97%. The authors concluded that the hybrid of FedAvg and cross-entropy approach was more effective compared to FedAvg alone, especially for clients that were either under-fitted or over-fitted.

v. Federated Learning for Microvasculature Segmentation and Diabetic Retinopathy Classification of OCT Data

Lo *et al*., in 2021 [92], proposed a federated learning-based model for RDR classification and compared the efficiency of the FL model with the standard models for segmenting microvasculature and binary class classification of RDR and Non-RDR images. FL models were simulated using the OwnCloud platform. The VGG19 technique, coupled with ImageNet weights, was utilized for extracting features, and 2 fully connected layers were used for classification. The data were acquired from various medical institutions. The segmentation was performed on a sample size of 153 OCTA images, which were manually labelled by retinal experts. The dual-class classification into RDR and non-RDR was done on 700 images. The non-RDR images numbered 337, while the RDR cases totaled 363. The results obtained were similar in both the decentralized federated and centralized standard models for segmentation and classification. The authors found that the mean DSC was 0.807 for the centralized dataset, and for the federated dataset, it was 0.793. The federated models used for the classification yielded an average AUROC of 0.954 and 0.960, while the internal models in the centralized system scored a mean AUROC of 0.956 and 0.973, respectively. The authors concluded that the results of FL are comparable to those of the standard centralized model, and the accumulation of the dataset at a central location does not offer many benefits.

vi. Collaborative Differentially Private Federated Learning Framework for the Prediction of Diabetic Retinopathy

Hossain *et al*., in 2023 [93], introduced a model utilizing collaborative, differentially private FL specifically designed to predict the severity of DR diseases. This model was trained on a dataset with 35,120 retinal images, which are categorized into five distinct classes. The researchers worked on several prominent DL algorithms, including AlexNet, ResNet50, SqueezeNet1.1, and VGG16, to evaluate their effectiveness in the FL context. Among these, the ResNet50 achieved the highest accuracy of 83.05% under standard conditions and 79.35% when a noise multiplier of 8.0 was applied. The authors concluded that the method significantly reduced communication overhead by 49% compared to traditional FL approaches. The achieved efficiency was indicative of the balance between ensuring data privacy and maintaining a high level of model accuracy.

vii. CD-FL: Cataract Images-Based Disease Detection Using Federated Learning

Khan *et al*., in 2023 [94], proposed a privacy-preserving federated learning framework, called CD-FL, for detecting cataracts using fundus images. The FL framework used the VGG16 deep neural network at the backbone for training clients and the server. The dataset used was the Ocular Disease Intelligent Recognition (ODIR) dataset, containing 10,000 fundus images from eight different categories. For cataract and normal eyes, 6,392 training images were used out of all the images. The overall accuracy was around 95.28%. The researchers used only 2 clients for training, which is a limited number. Thus, performance with more clients in larger networks remains largely unexplored. The future investigations need to apply advanced DL algorithms to improve accuracy and utilize multiple datasets. Additionally, utilizing statistical tests to validate the quality is also required.

viii. FedDL: Personalized Federated Deep Learning for Enhanced Detection and Classification of Diabetic Retinopathy

Bhulakshmi *et al*., in 2024 [95], developed an FL framework for preserving data privacy while enhancing the overall accuracy of DR detection. Two open-source datasets were used by the researchers – the IDRiD dataset with 516 retinal high-quality fundus images for training and validation purposes, and the Structured Analysis of the Retina (STARE) dataset with 400 images for generalization of the model. Five of the most coveted DL-based CNN models were used and compared to achieve optimal performance. These models were ResNet50, DenseNet201, AlexNet, EfficientNetB7, and VGGNet19. The highest performance was for ResNet50, achieving an accuracy score of 94.66%. The aggregation method used was the Federated Averaging (FedAvg). The future directions include exploring the impact of heterogeneous distribution and also investigating advanced aggregation techniques and privacy-preserving methods, such as differential privacy.

ix. A Privacy-Preserving Collaborative Federated Learning Framework for Detecting Retinal Diseases

Gulati *et al*., in 2024 [23], proposed a privacy-preserving federated deep learning (FDL) framework for the detection of diabetic retinopathy (DR) and diabetic macular edema (DME). The framework was designed with a lightweight MobileNetV2 architecture for efficiency. The implementation compared two aggregation strategies, FedAvg and FedProx, in IID and non-IID data scenarios. The dataset used in the study was pooled from two different open-source datasets: Eye Fundus and OCT images of the Translational Visual Health Laboratory repository and the Indian Diabetic Retinopathy Image Dataset (IDRiD) from Kaggle. The FDL framework with MobileNetV2 used for detecting DR and DME used Adam optimizer with a learning rate of 0.001 and achieved 98.69% accuracy for FedAvg with IID data and 87.09% accuracy with non-IID data; while for FedProx, the accuracy achieved with IID data was 98.03% and with non-IID was 98.25%.

x. Collaborative, Privacy-Preserving Federated Learning Framework for the Detection of Diabetic Eye Diseases

Gulati *et al*. (2024) [20] introduced a Federated Deep Learning (FDL) framework for detecting diabetic eye diseases (DEDs), such as Diabetic Macular Edema (DME), Diabetic Retinopathy (DR), and glaucoma. The focus was to ensure data privacy while maintaining high diagnostic accuracy and model stability. The authors utilized MobileNetV2, a lightweight and efficient DL model at the backbone for training, and the Federated Proximal (FedProx) aggregation strategy was used to mitigate the impact of heterogeneous client data. A total of 2700 high-resolution colored fundus images across four classes- DME, DR, glaucoma, and normal eyes, sourced from three different open-source datasets, namely the OCT and Eye Fundus Dataset, the Indian Diabetic Retinopathy Image Dataset (IDRiD), and the Ocular Disease Intelligent Recognition (ODIR) dataset, were used. The performance of the multi-class classification FL framework was very high with 98.7% accuracy. In the future, communication protocols can be optimized to minimize overhead and delays.

xi. Enhanced Privacy-Preserving Architecture for Fundus Disease Diagnosis with Federated Learning

Jiang *et al*., in 2025 [96], introduced a DataWeightedFed, a federated learning framework for fundus disease diagnosis. The method combined random k-client selection and a weighted FedAvg aggregation to preserve patient privacy while narrowing the accuracy gap with centralized training to just 1.85%. The evaluation of their method was conducted on a subset of the ODIR dataset, comprising three classes and four clients. The VGG-19 backbone model was used, with frozen convolutional layers and a transfer-learned classifier head. However, the scope of the article is limited by its small client count and narrow range of disease categories. It also lacks concrete differential-privacy parameters or communication-cost analysis and relies on a heavy backbone model.

xii. Privacy-Preserving and Collaborative Federated Learning Model for the Detection of Ocular Diseases

Gulati *et al*. [97] proposed a horizontal federated learning (FL) framework for the detection of diabetic retinopathy (DR) and diabetic macular edema (DME) from fundus images while preserving patient privacy. The backbone model used was MobileNetV2, and they simulated FL with 2, 3, and 4 clients on a composite Kaggle dataset of 838 fundus images (278 DME, 280 DR, 280 normal). Data were augmented (rotations, flips), normalised to ImageNet statistics, and split 80:20 for training/testing. Clients were trained locally for five epochs per round and over 30 communication rounds, sharing only model updates aggregated *via* FedAvg on the server. Across scenarios, the FL system achieved an average accuracy of 96% and class-wise accuracy of 100% for both DR and DME. The study utilized a small dataset of 838 images from just two diseases, making it unclear how well the model would perform on larger, more diverse real-world data. It also relies on the basic FedAvg method to combine updates and does not explore stronger or more privacy-focused options, such as FedProx or secure aggregation.

Table **3** provides an overview of the objectives, methodologies, datasets, and key findings of each study, highlighting their unique contributions to the field of ocular disease detection using FL and AI techniques.

#### 2.4.3 Literature Review using Federated Learning Models in Disease Detection

This section offers a literature review on the application of FL models for disease detection, highlighting research trends and findings in this emerging field.

i. Federated Learning for Breast Density Classification: A Real-World Implementation

Roth *et al*. (2020) [98] presented a distributed system incorporating FL, where FedAvg was used as the aggregation technique for updating the global model. A mini-batch with a size of 32 was used to select participating clients for the current round. The implementation was done using TensorFlow and NVIDIA Clara Train SDK. The images were first normalized and resampled, and augmentation was applied before training to prevent overfitting of the model. The global model was trained using 300 rounds of training at clients and aggregation at the server. The dataset used included real-world data from seven different medical organizations, consisting of mammographic images. The dataset was non-IID and heterogeneous, containing a total of 142952 images. The distribution of breast density classes varied significantly, with some areas being entirely fatty, extremely dense, or heterogeneously dense. The evaluation of the model was conducted using Cohen’s Linear Weighted Kappa score, taken both prior to and after applying Federation Models, as well as after the fine-tuning round. The performance improved by 6.3% on average while the training was conducted in a distributed manner, rather than at each individual’s local site. Additionally, a 45.8% improvement was observed in the generalizability of the models. In conclusion, the federated learned models demonstrated comparable performance, as mentioned in the literature, to centralized implementations.

ii. Dynamic-Fusion-Based Federated Learning for COVID-19 Detection

Zhang *et al*., in 2021 [99], proposed a dynamic fusion-based federated learning model for detecting COVID-19. The model used 2 decision-makers: Client Participation and Client Selection. Participation was decided by each client as to whether they should join the current round and update the central system henceforth. Two types of images were included in the experimental setup: first, CT images, and second, X-ray images. The CT image set was an accumulation of 746 images; 349 were COVID-positive and 397 were diagnosed negative. The X-ray set consisted of a total of 2960 images, of which only 274 were COVID-positive, 1341 were negative, and 1345 were viral pneumonia cases. From a total of 3706 images, 3326 images were selected. Of these, 2800 were used for training the algorithm, and the remaining 526 images were used for the test cases. Three different models were used: ResNet50, ResNet101, and GhostNet. The accuracy of the three models was compared for various partitions of the dataset, and the highest accuracy was achieved for the last partition with 91.08% for GhostNet, 95% for ResNet50, and 97.5% for ResNet101. Thus, ResNet101 performed best for the dynamic fusion-based FL technique. The authors concluded that the approach followed yields a better accuracy score but takes more time for training than the default federation model.

iii. Federated Semi-Supervised Learning for COVID Region Segmentation in Chest CT Using Multi-National Data from China, Italy, Japan

A federated learning model was developed by Yang *et al*. in 2021 [100] for classifying 3D chest x-ray (CXR) images for COVID-19 detection, addressing data heterogeneity and privacy through FL. The authors employed semi-supervised learning at the global level. The employed technique utilized datasets from different machines and used both labeled and unlabeled datasets in client models, with partial global model updates based on a loss function. The dataset used was from three hospitals in China, Italy, and Japan, comprising 1704 chest CT scans, with 945 manually evaluated by radiologists. The authors conducted an ablation study and assessed performance using Dice’s score. They found that frequent aggregation improved the performance of self-supervised models, while it led to performance deterioration in supervised models. The authors developed a CNN model comprising five convolutional layers and two fully connected layers, utilizing ReLU and Softmax functions. The global parameter aggregation used was FedAvg, with communication efficiency achieved by weight pruning and quantization. The dataset, a combination of five public sources, included 28833 X-ray images categorized into COVID-19, pneumonia, lung opacity, and normal. The dataset was split 90:10 for training and testing. Model accuracy was evaluated, with the standard central model achieving 93.44%, the federated model 92.26%, and the proposed communication-efficient model 92.24%. The authors concluded that the proposed model's efficacy was comparable to others, but its speed was about ten times faster.

iv. A Federated Learning Approach to Pneumonia Detection

Khan *et al*., in 2021 [101], proposed a federated deep-learning model for pneumonia detection using chest X-ray images. The authors identified foggy regions as regions of interest in the lung. The dataset consisted of chest X-ray images of subjects with pneumonia and COVID-19, as well as some images from non-sufferers. The detection of pneumonia was the subject of the study, so images from patients with COVID-19 were discarded, leaving 5856 images. The images were split into two sets: 4684 training images and the remaining 1172 images, which were testing images. The raw images were first pre-processed by applying a Canny filter to the dataset, and then FL was applied. The dataset was from two hospitals, so two client simulations were done, each representative of the two hospitals.

The training of the models was done using four DL methods: a CNN model, a ResNet50 model, a VGG16 model, and an AlexNet model. The CNN model used had four convolutional layers and four max-pooling layers, along with two dense layers for training. The global models were aggregated with the local models multiple times to fine-tune them, and the performance was estimated based on accuracy, recall, precision, F1-score, Cohen’s Kappa, AUROC, sensitivity, and specificity scores. The authors found that the most suitable model was VGG16, with an accuracy of 0.91, followed by CNN with an accuracy of 0.74. ResNet50 and AlexNet both demonstrated an accuracy score of 0.73. The precision, recall, F1-score, AUROC, specificity, sensitivity, and Cohen’s Kappa scores obtained by the VGG16 model were 0.94, 0.93, 0.93, 0.89, 0.92, 0.85, and 0.77, respectively.

v. Investigating Federated Learning Strategies for Pneumonia Image Classification

Balaji *et al*., in 2021 [102], proposed a federated learning model for the classification of pneumonia on chest X-ray images. The researchers evaluated the proposed FL model using three aggregation strategies and compared their performance with that of the centralized model. The three strategies used were FedAvg, COMED (Coordinate-wise Median), and GEOMED (Geometric Median). Additionally, a Secure Multiparty Computation (SMPC) layer with a shuffling policy was added to boost data privacy. The dataset was the ImageNet-1K dataset with a total of 5856 CXR images categorized into normal and pneumonia classes. The test set contained 390 pneumonia images, and 234 were normal, while the training set contained 1341 normal images and 3875 pneumonia images. The federating learning model had two servers: one was the SMPC server, and the other was the aggregation server. In this technique, clients underwent training and communicated the layers to the SMPC server, which applied a shuffling algorithm. The shuffled layers were then sent for aggregation at the aggregating server. The aggregating server then either averaged the values using the FedAvg function or employed a COMED or GEOMED. COMED was a median strategy for resisting changes to the models due to anomaly data. In this process, the aggregator first implemented sorting on the parametric values of all the clients and then took the median value as the optimized value. In the GEOMED model, aggregation was assumed to be a convex averaging function with just a single global minimum. The model was updated until a global minimum was achieved, which is the geometric median of the received models. Four CNN models were used for training: AlexNet, ConvNet, ResNet18, and ResNet50, and a comparative analysis was carried out in three scenarios with aggregation as FedAvg, GEOMED, and COMED. The authors concluded that COMED achieved 31.2% more accuracy in comparison to the standard model, and GEOMED achieved a 30.4% better accuracy score than FedAvg.

vi. Blockchain-Federated-Learning and Deep Learning Models for COVID-19 Detection Using CT Imaging

Kumar *et al*., in 2021 [103], designed a collaborative model using FL for detecting COVID-19 disease using computed tomography scan images of subjects. The dataset was procured locally from 3 different hospitals and consisted of 34,006 CT images. The proposed model involved several steps: first, the data were normalized for uniformity, and then DL models were utilized to identify patterns from the CT scan images of the lung. The image segmentation technique used was SegCaps, and the network training was done using a Capsule Network similar to an Artificial Neural Network. The concept of FL was applied thereafter. Precision, sensitivity (recall), specificity, and accuracy were used to evaluate and contrast the model with other popular DL techniques and the proposed method. The proposed method obtained an accuracy of 98.68%, while the precision and sensitivity scores were 0.830 and 0.987, respectively, and the specificity score was 0.004.

vii. FedRare: Federated Learning with Intra- and Inter-Client Contrast for Effective Rare Disease Classification

Wu *et al*. (2022) [104] proposed the FedRare method for identifying rare diseases that suffer from class imbalance. The authors proposed the FL model to address data heterogeneity by extracting separable latent features for classification tasks, utilizing intra-client learning and contrastive supervised learning. The authors extracted and selected reliable latent features at the central server and then provided the features to the clients as feedback. A 10-client setup with 6 diseased clients and 4 normal clients was simulated. The FedRare model exhibited an upsurge of 9.6% and 5.9% in balanced accuracy in comparison to the FedAvg and FedIRM base models. The dataset included samples of seven diseases, out of which two were rare diseases (DF and VASC) with the least number of samples. The official dataset consisted of a total of 11,720 images, which were divided into a training set, a validation set, and a testing set, containing 10,015, 193, and 1,512 images, respectively. There were 7011 images in the training set and 3004 images in the validation set with a 70:30 ratio. The local training at the client end utilized the contrastive learning concept for intra-client learning and inter-client learning. The server aggregation required all the identified latent features to be aggregated, and FedAvg was used at the server for that purpose. The performance of the proposed FedRare model was compared with that of FedAvg, FedProc, MOON, FedProx, and FedIRM using Balanced Accuracy (B-ACC) as the evaluation metric. The authors concluded that the average B-ACC of the FedRare model was the highest at 79.7%, followed by FedIRM, MOON, FedProx, and FedAvg at 73.8%, 73.1%, 71.7%, and 70.1%, respectively, with the lowest score of FedProc at 66.8%.

viii. SplitAVG: A Heterogeneity-Aware Federated Deep Learning Method for Medical Imaging

Zhang *et al*., in 2022 [105], proposed a heterogeneity-aware method to enhance the performance of federated learning models in the case of data heterogeneity. The researchers employed three distinct datasets, including a comprehensive collection of 44,351 retinal images from the Kaggle Diabetic Retinopathy Competition, 14,236 pediatric radiographs from Kaggle's BoneAge set, and a third dataset manually gathered from various institutions, comprising images from 165 subjects. The authors compared their proposed SplitAvg method with seven prominent FL methods, focusing on the efficacy of the model. The SplitAvg method demonstrated superior performance, with 96.2% accuracy. The researchers concluded that the SplitAvg method is potentially effective in training models on heterogeneous datasets.

ix. Integrated CNN and Federated Learning for COVID-19 Detection on Chest X-Ray Images.

Li *et al*., in 2022 [106], proposed an aggregation model, FedFocus, with a dynamic focus for the detection of COVID-19 disease in the CXR images. The dataset was curated from multiple open-source images of chest radiography and named COVIDX8. The collection comprised 16689 images of chest X-rays, and 1769 of them were COVID-19 specimens, 6069 were pneumonia samples, and 8851 were normal samples. These images were collected from 3 different cities in China: Wuhan, Beijing, and Hong Kong. The model proposed was based on a weight loss-dynamic tuning parameter, which had to be updated after every round of client training. The global parameters at the server were updated based on this tuning parameter, unlike FedAvg, which is just an averaging function. The aggregation algorithm, known as FedFocus, was applied to the central server during parameter updates of the global model. Both the central and the local training used ResNet-18 for training purposes. The authors concluded that their proposed model displayed a superior performance in comparison to the base models in terms of accuracy, efficiency, and stability. The accuracy of FedFocus was highest in the case of COVID-19 detection, with 65.24%, 93.29%, and 96.34%, respectively. In contrast, in the case of pneumonia, the prediction accuracy for FedAvg was higher, with 81.91% for standard FL, 88.65% for FedAvg, and 86.18% for FedFocus.

x. A Transfer Learning Approach to Breast Cancer Classification in a Federated Learning Framework

Tan *et al*., in 2023 [107], proposed an FL framework combined with transfer learning techniques for breast cancer classification. The FL framework employed a FeAvg-CNN and a MobileNet-based approach to counter data heterogeneity, privacy concerns, and limited dataset sizes. The proposed work was based on pretrained CNNs for enhancing feature extraction and employing synthetic minority oversampling techniques (SMOTE) to improve the classification performance of imbalanced datasets. The dataset used in the FL implementation was the Digital Database for Screening Mammography (DDSM), specifically the curated subset known as CBIS-DDSM. The dataset was curated by trained mammologists and was a collection of 2,620 scanned film mammography studies, including normal, benign, and malignant cases. The efficacy of the proposed model was nearly 98%, outperforming centralized learning methods.

xi. Secure Federated Learning for Parkinson’s Disease: Non-IID Data Partitioning and Homomorphic Encryption Strategies

Tanim *et al*. (2024) [49] introduced an FL framework focused on tackling data heterogeneity, *i.e.*, non-IID data, and enhancing privacy with homomorphic encryption. A clinical dataset focused on Parkinson’s disease, called the PD-BioStampRC21 dataset, was used to study the progression of Parkinson's disease and its motor symptoms. The proposed partitioning scheme securely aggregated the model updates, employing Cheon-Kim-Kim-Song (CKKS) encryption to protect sensitive data and ensure compliance with privacy regulations.

xii. Secure FL for Alzheimer's Disease Detection

In an article by Mitrovska *et al*. (2024) [108], a FL framework for Alzheimer's disease detection using structural MRI data was proposed. The authors integrated Federated Averaging and Secure Aggregation in the FL model. The dataset employed was the Alzheimer's Disease Neuroimaging Initiative (ADNI), comprising 618 subjects, including 166 patients with Alzheimer's disease and 452 cognitively normal controls. A 3D convolutional neural network was used to train each client, sending encrypted updates to a central server for secure aggregation. The performance was tested with imbalanced data across clients. The authors demonstrated that federated models with secure aggregation achieved accuracy close to centralized training while protecting privacy; however, performance decreased when the client data was significantly different. Therefore, strategies for non-IID settings, which are a usual scenario practically, such as FedProx, have not been explored.

##### 2.4.3.1 Enhancing Heart Disease Prediction with FL and Blockchain Integration

Otoum *et al*., in 2024 [109], proposed a system for predicting heart disease using FL with the TabNet model and blockchain. The proposed method was tested on two datasets: the comprehensive heart disease dataset and the UCI heart disease dataset. The aggregation was performed using the FedAvg algorithm, integrated with Blockchain to maintain a secure record of model updates. The results showed that the model achieved approximately 82% accuracy after 50 training rounds with a specific privacy setting. However, the proposed work has some limitations, including the need for additional computing time for blockchain and weaker results on smaller datasets, such as the UCI dataset, which can lead to overfitting.

Table **4** presents a summary of the works presented for the detection of various diseases using FL models.

## 3 RESULTS AND DISCUSSION

This sub-section provides insights into the use of models in detecting eye disease and discusses existing research with its shortcomings and future scope.

### 3.1 A Detailed Analysis of Ocular Disease Detection Trained through Machine Learning Models

Rekhi *et al*. (2017) [74] proposed an automated detection system for grading diabetic macular edema using colored fundus images. The macula was detected using the morphological features in the fundus images. The segmentation was done using the Support Vector ML Algorithm. The authors relied on quite small databases (DIARETDB1 and MESSIDOR), which may not represent all variations in DME cases globally and may also lead to overfitting. Therefore, expanding the work to incorporate more diverse datasets could improve the algorithm's robustness. The algorithm demonstrated good prediction accuracy; however, its performance in a clinical setting, where it would be dealing with a broader range of images and conditions, has not been thoroughly assessed. Accuracy is of utmost importance in medical data. In addition, the authors did not delve deeply into the computational efficiency or the algorithmic complexity, which is crucial for real-time applications. A more comprehensive comparison with existing methods could better position the proposed method within the current landscape of DME detection technologies. The algorithm's improved adaptability to diverse clinical environments is also not addressed.

Marin *et al*. (2018) [75] developed a system to identify the risk of DME using fundus photographs. The detection of exudates in these images is crucial for early disease identification. The system employed supervised learning techniques in its learning process and achieved a specificity score of approximately 70% and a sensitivity of approximately 90%. The results suggest that further training and tuning hyperparameters could enhance the system's performance. The image dataset was acquired from a public database called the MESSIDOR. However, a detailed analysis of computational efficiency and scalability for real-time applications is lacking. There is a need for a more comprehensive comparison with other existing disease-specific detection methods. In this study, the system's ability to generalize to other disease conditions was not explored. The scalability of the method to a higher set of images also needs to be explored further.

Selvathi *et al*. (2019) [79] proposed a method for automatically detecting diabetic eye diseases using thermographic images. After preprocessing, the images were classified using a supervised support vector ML algorithm. The binary classification, which differentiated between healthy and diseased eyes, achieved an accuracy of 86.22%. The model obtained a specificity score of 79.17% and a sensitivity score of 94.07%. This work utilized a dataset of 283 thermal images, comprising 149 images from individuals with diabetes and 134 from healthy individuals. Hence, the dataset is quite limited, which affects the model's robustness and generalizability. The use of thermal images for detecting eye disease is infrequent, which also becomes a limiting factor for the practical application of this method. The authors did not address the integration of their method into existing clinical workflows. The performance of the system is not as good as required in medical applications, such as disease detection, and lags behind other proposed methods in the literature.

### 3.2 An Analytical Discussion on Ocular Disease Detection Models Trained through Deep Learning

This sub-section critically analyzes deep learning models used for the detection of diabetic eye diseases while discussing the gaps and shortcomings.

Qummar *et al*., in 2019 [76], developed a model for classifying DR into five stages: normal stage, mild stage, moderate stage, severe stage, and proliferative stage, using a Deep CNN. They utilized five CNN models: DenseNet-121, DenseNet-169, ResNet-50, Xception, and Inception-V3 to enhance classification accuracy. The model was trained on a dataset of 35,126 color fundus images from Kaggle, representing the different severity stages of DR. An ensemble approach combining these CNN models was used to improve the classification. Although the dataset was large enough, the model presented in the work achieved an accuracy of 80.8%, which is not considered satisfactory in healthcare applications. The dataset is imbalanced, with varying numbers of samples for each class; therefore, the accuracy may be biased. To remove this bias, down-sampling and upscaling have been performed, and the accuracy of the upsampled data improved considerably; yet, there is still much scope for improvement. The study relies on a single dataset, which may be a limiting factor for the generalizability of the work. Additionally, since the ensemble utilizes five other models, it is quite complex and computationally intensive, making practical deployment a challenge.

Zeng *et al*. (2019) [77] developed an innovative method for automatically detecting RDR using fundus photographs. They designed an architecture resembling a Siamese network and employed deep CNNs for learning. The dataset, sourced from the Kaggle diabetic retinopathy competition provided by EyePACS, included 35,126 high-resolution fundus images captured under various conditions. The evaluation was based on a kappa score of approximately 0.829, which needs to be significantly improved. The dataset was quite large, and the algorithm was also quite complex, which might result in overfitting of the data. Additionally, the sophisticated architecture proposed in this work would require high computational resources, potentially limiting its accessibility in resource-constrained environments. The study also lacks extensive validation in real-world clinical settings, which is pivotal for the practical deployment of the proposed work.

Aamir *et al*. (2020) [78] introduced a method for detecting and classifying glaucoma into various stages, including early, advanced, and moderate, using a multilevel and deep CNN. The authors utilized deep CNNs to detect and perform multi-label classification on fluorescence fundus angiography (FFA) images. Although the approach achieved impressive results, the complexity of the ML-DCNN could pose challenges in terms of computational efficiency for practical applications. The exploration of the model's integration and performance in real-world clinical settings is yet to be monitored. Additionally, testing is necessary to prevent the model from overfitting. There could also be potential bias due to an imbalanced dataset, with a significant majority of images belonging to the 'normal' category. There is limited validation, as the study primarily focuses on a dataset from a specific geographic region, which might not represent the global population. The authors created their own dataset at the Eye Center of Zhejiang University, comprising 4,067 images to identify four types of lesions: laser scars, non-perfusion regions, leakages, and microaneurysms, using three different CNN models, namely VGG16, DenseNet, and ResNet50. Among these, DenseNet showed the most effective performance in identifying these lesions. The specificity and sensitivity of the model for some lesion types were lower compared to manual classification by ophthalmologists, indicating areas for improvement. The dataset was imbalanced, with varying numbers of samples in each class, suggesting the possibility of overfitting.

Jiang *et al*. (2020) [81] proposed a deep learning model for detecting glaucoma in diabetic patients by jointly segmenting the optic disc and optic cup using a Joint RCNN. The research utilized datasets from the Singapore Chinese Eye Study and ORIGA, comprising 650 and 1,676 images, respectively. The approach yielded improved results, with AUC values of 0.901 on the SCES dataset and 0.854 on ORIGA. However, the primary focus was on glaucoma detection, which limits the model's application to other eye diseases without modification and retraining. The validation and detailed performance metrics are essential for assessing the clinical applicability and efficacy of the model, which are not provided in the study.

de la Torre *et al*. (2020) [82] introduced a DL model to classify DR into different severity levels. The proposed work utilized a score propagation model to assess the severity level of retinopathy in the patient’s eyes. The study used a dataset from EyePACS on Kaggle, comprising 75,650 images for training the model and 10,000 high-quality images for testing. The model demonstrated an accuracy of 85.7% (0.857) and 91.0% (0.910), showing promise in classifying DR based on severity, yet not reaching the benchmark of the state-of-the-art models. The dataset is quite large, and the proposed technique is quite complex, which may result in model overfitting.

Sarki *et al*. (2020) [83] highlighted the significance of timely diagnosis of diabetic eye diseases (DEDs), such as diabetic retinopathy (DR) and diabetic macular edema (DME), in preventing permanent vision impairment. The manual screening of these DEDs is effective but resource-intensive. Therefore, automatic detection systems are required to shorten the detection time. The authors address this gap by utilizing deep learning (DL) to automate detection, with a focus on mild and multi-class classification, where subtle features often pose significant challenges. The authors leverage transfer learning models, VGG16 and InceptionV3, which are pre-trained on ImageNet, and fine-tune these models for fundus image datasets, including publicly available repositories, such as MESSIDOR and DRISHTI-GS. The approach lacks the ability to distinguish early-stage lesions, highlighting the potential for integrating lesion segmentation techniques in future research. The research relies on small, open-source datasets that may limit generalizability. Additionally, the drop in performance with increased classification categories indicates a need for domain-specific enhancements.

Karimi *et al*. (2024) [84] explored the use of clinical, lifestyle, and demographic data from Electronic Health Records (EHR) combined with machine learning for glaucoma detection. The authors utilized models, such as Random Forest, Gradient Boosting, and TensorFlow Sequential, for predictive analytics in ophthalmology. The Random Forest performed best with 67.5% accuracy, highlighting its strength in structured data over imaging-based approaches. The findings suggest that factors, such as intraocular pressure (IOP), family history, and body mass index (BMI), play a significant role in the progression of the disease. The dataset was biased and imbalanced, containing a disproportionate number of samples from a single community, which limited the model's generalizability across diverse demographics. The EHR-based models offer valuable insights but demonstrate lower accuracy compared to imaging-focused methods, such as OCT or fundus photography.

Gao *et al*. (2024) [85] presented a Dual-Stream Cataract Evaluation Network (DCEN) for the classification and severity grading of cataracts using deep learning and fundus images. The designed model enables simultaneous classification and grading of the disease, demonstrating that combining features can enhance the effectiveness of DL models. The approach delivered good results, achieving 97.62% accuracy for type classification and 97.03% for severity grading. However, the proposed model is limited due to imbalanced data distribution, with a very small number of cases of posterior subcapsular cataract (PSC) compared to other types, which may reduce its effectiveness for rarer conditions. Additionally, the images used in the dataset are acquired using the same equipment, which limits the model’s adaptability to heterogeneous data from different devices. The dataset used in the work also lacks demographic details, such as age and sex, which may introduce bias. The future directions include expanding and diversifying datasets, incorporating demographic data, and reducing bias while enhancing practical utility.

Elkholy *et al*. (2024) [86] focused on detecting ophthalmic diseases like Diabetic Macular Edema (DME), Choroidal Neovascular Membranes (CNM), and Age-related Macular Degeneration (AMD) by leveraging CNNS. The authors utilized transfer learning and fine-tuned pre-trained models, such as VGG16, achieving a classification accuracy of 97%. The proposed method demonstrated superior performance; however, it suffered from some issues, such as the dataset being balanced in all aspects, which is impractical and may lead to model overfitting. The work relies on high-quality images, whereas the images are actually from different sources, which might reduce performance. In the future, the interpretation of OCT images can be enhanced by integrating advanced segmentation techniques, leading to a more accurate analysis of lesions.

Deepak *et al*. (2024) [87] utilized DL-based models to detect two ocular diseases, cataracts and glaucoma, in a timely manner. The authors employed three optimized CNN models, SqueezeNet, DarkNet53, and EfficientNetB0, and experimented with different batch sizes (6, 8, 10) and optimizers (SGDM, RMSProp, and Adam). The best performance was achieved with DarkNet-53, which scored an accuracy of 99.4%. The ODIR dataset, although real-world, contains limited disease categories, potentially restricting the model's generalizability to other ocular conditions. The model relies on high-resolution fundus images, which may not reflect the variability in image quality encountered in real clinical settings. In the future, techniques, such as noise reduction and color correction, can be incorporated to enhance robustness across diverse imaging environments.

Jiang *et al*. (2025) [96] proposed a federated learning (FL) framework for fundus disease diagnosis using VGG-19 and a weighted aggregation method (wFedAvg). The model achieved an accuracy of 84.88%, with only a 1.85% reduction from centralized training, demonstrating significant improvement over traditional FL methods. However, there are some limitations; the model was tested with only four clients and on a subset of diseases (normal, glaucoma, and cataract), which limits its scalability and generalizability. Moreover, balanced data distribution and reliance on high-quality, pre-processed images may not reflect real-world clinical variability. The impact of differential privacy on model utility was also not deeply examined. Future work should test scalability with more clients, extend to broader disease categories, and evaluate performance under non-IID and lower-quality image conditions to enhance real-world applicability.

Gulati *et al*. (2025) [97] demonstrated an FL framework using MobileNetV2 and FedAvg for diagnosing diabetic retinopathy and diabetic macular edema. The model was simulated using 2, 3, and 4 clients. The model achieved an overall accuracy score of 96% and a class-wise accuracy score of 100% for both diseases, while preserving data privacy. However, the study utilized a small, curated dataset (838 images) sourced from two Kaggle datasets with a balanced class distribution, which may limit its real-world applicability. Additionally, performance consistency began to decline slightly with 4-client setups, highlighting potential data complexity and scalability challenges. In the future, exploring generalization with non-IID larger datasets and integrating differential privacy for stronger security guarantees should be considered.

An analytical summary of the ML and DL models used in detecting DR and DME is given in Table **5**.

### 3.3 An Analytical Discussion on Ocular Disease Detection Models Trained through Federated Learning

This subsection investigates the application of FL models in identifying eye diseases and highlights their limitations.

Matta *et al*., in 2023 [88], developed a collaborative model for categorizing images into normal and RDR stages. The EfficientNet-B5 algorithm was employed for server and client module learning. The model utilized weighted and unweighted averaging for client-server data aggregation; other aggregation strategies have not been explored. Utilizing the OPHDIAT dataset, the study involved analyzing 697,275 images from 5 to 43 clients, which might result in network and communication overhead. The performance was assessed using the AUC, showing that the federated models' effectiveness was comparable to that of centralized DL methods. However, performance metrics, such as prediction accuracy, precision, recall, sensitivity, and F1-score, which are essential for a detailed comparison, were not provided. The privacy and security concerns were not addressed, which is necessary for the system to be consistent and for sensitive data to be secured on the network. The large dataset will increase the server's load in terms of computational requirements and memory management. In this scenario, client selection and weight pruning are required.

Tang *et al*., in 2023 [89], devised an FL approach with 5 clients for the multiclass classification of seven eye diseases: glaucoma, DR, myopia, cataract, age-related macular degeneration (AMD), hypertension, and others, prioritizing data privacy enhancement. A two-phase Gaussian randomization process was employed to secure data privacy. The approach also integrated domain confusion and multi-expert learning to maintain data confidentiality while addressing domain-gap challenges. The OIAODIR dataset, comprising anonymized data from 5,000 patients, was used; however, the class division was not provided, and no methods to address class imbalance were mentioned. Performance metrics included the AUC and the F1-score, which were not sufficient to analyze the performance of the model, and also, the metrics obtained were insufficient for healthcare applications. Therefore, the performance of the model needs significant improvement. Additionally, other aggregation strategies, such as FedProx and FedSVRG, were not explored. Important model parameters, such as learning rate, batch size, number of epochs, and communication rounds, were also not specified.

Nasajpour *et al*., in 2022 [90], focused on the detection of DR, a serious vision-threatening disease, and employed a collaborative FL model for the automatic detection and grading. The data for this study were sourced from five different public DR datasets: EyePACS, MESSIDOR, University of Auckland (UoA), APTOS, and IDRID, totaling around 3,000 images. The research explored two different aggregation strategies in FL, FedAvg and FedProx, and compared their performance with a centralized DL model. The AlexNet model was used for training both the central model and the local models at the client sites. The researchers concluded that the accuracy of the federated models was comparable to that of the standard model, with the standard model achieving 92.19% accuracy. The federated model with FedAvg aggregation achieved 90.07% accuracy, while FedProx aggregation yielded 85.81%. The model parameters, such as the number of clients, communication rounds, learning rate, epochs, and batch size, which are essential for evaluating and comparative analysis of an FL scenario, were not mentioned. Furthermore, efficient communication strategies and privacy-preserving techniques for data transmission over the network were also not explored.

Mohan *et al*. (2023) [91] developed a CNN-based model for detecting DR by analyzing fundus images. The model incorporated local, global, and intermediate image features and was tested across diverse client databases. It integrated the FedAvg method with median cross-entropy, enhancing its effectiveness for both under-fitted and over-fitted clients. However, the use of efficient communication strategies was not explored. The dataset, comprising 5,000 images sourced from MESSIDOR-2, IDRID, Kaggle, and a local hospital, was evenly distributed among five clients. However, the data is IID, whereas a realistic federated learning (FL) setup typically involves non-IID data. The authors’ model achieved high accuracy using the FedAvg aggregation strategy with a learning rate of 0.01 and 20 communication rounds. However, alternative aggregation methods, such as FedProx, were not explored.

Lo *et al*. (2021) [92] proposed a federated learning model to classify RDR and non-RDR images. The effectiveness of FL models was compared to standard centralized models for segmenting microvasculature. The results showed that the FL scenario, which ensures data privacy, is as effective as centralized models. The proposed work utilized the VGG19 technique for feature extraction. The study analyzed 153 manually labeled OCTA images for segmentation and 700 images for RDR and non-RDR classification. Therefore, the dataset used was quite small and imbalanced. The performance metrics used included the DSC, 0.807 for the centralized model and 0.793 for the federated model, and the AUROC, which ranged from 0.954 to 0.960 for the federated model and from 0.956 to 0.973 for the centralized model, indicating comparable performance between the two approaches. However, other commonly used evaluation metrics, such as prediction accuracy, precision, recall, and F1-score, were not reported in detail. Furthermore, the study employed only an unweighted aggregation strategy, without analyzing or synthesizing other aggregation methods. Key aspects, such as privacy-preserving techniques (*e.g.*, Differential Privacy) and communication optimization strategies, were also not addressed.

Hossain *et al*., in 2023 [93], presented a differentially private FL model for predicting DR. It trained the FL model on 35,120 decentralized retinal images classified into five categories and used DL algorithms, AlexNet, ResNet50, SqueezeNet1.1, and VGG16, which were tested, with ResNet50 achieving the highest accuracy of 83.05% under standard conditions and 79.35% with a noise multiplier of 8.0. The aggregation was based on FedAvg, and the study did not explore other aggregation strategies. The model parameters, such as learning rate, epochs, batch size, and communication rounds, were not utilized. More importantly, a considerable trade-off between privacy and prediction accuracy occurred when applying differential privacy.

Khan *et al*. (2023) [94] focused on detecting cataract disease, a leading cause of vision impairment worldwide, and emphasized the importance of early detection in preventing blindness. The authors developed a privacy-preserving federated learning framework to address privacy concerns and data limitations in cataract detection from images, utilizing the VGG16 deep learning architecture. The privacy-preserving FL framework addresses privacy concerns and data limitations for detecting cataracts from images using the VGG16 deep learning architecture. Cataracts are a leading cause of vision impairment worldwide, affecting millions of people annually. Early diagnosis is crucial to prevent blindness, but privacy concerns and data limitations hinder the application of traditional artificial intelligence (AI) methods. To address these challenges, a study introduced a privacy-preserving approach using Federated Learning (FL) and the VGG16 deep neural network. The introduced model achieved an overall accuracy of 95.28%, a commendable performance score. However, challenges, such as communication overhead, data pre-processing inefficiencies, and potential overfitting in FL networks, have been identified. In the future, these limitations can be addressed by utilizing enhanced model architectures and optimization algorithms to facilitate wider adoption.

Bhulakshmi *et al*., in 2024 [95], leveraged FL for DR stage classification and trained machine learning models on decentralized data, ensuring patient privacy by sharing only model parameters instead of raw data. Five deep learning models, including ResNet50, DenseNet201, AlexNet, EfficientNetB7, and VGGNet19, were implemented within the FL framework, with ResNet50 achieving the highest accuracy of 94.66% on the IDRiD dataset. However, the dataset was quite small due to class imbalance, and the FedAvg algorithm was used, assuming a uniform class distribution. Therefore, the dataset needs to be pooled to build more effective FL models, and other communication strategies should be employed to handle class imbalance efficiently. Additional datasets must be explored to enhance model capabilities, and other aggregation strategies like FedProx and privacy-preserving techniques like differential privacy must be integrated.

Gulati *et al*., in 2024 [23], addressed an important problem of detection of vision-threatening retinal diseases like Diabetic Retinopathy (DR) and Diabetic Macular Edema (DME). The use of federated learning with MobileNetV2 DL architecture and FedAvg and FedProx aggregation strategies provides a framework for collaborative model training while preserving data privacy and detecting retinal diseases. The dataset was compiled from two different sources: Eye Fundus and OCT images from the Translational Visual Health Laboratory repository, and the Indian Diabetic Retinopathy Image Dataset (IDRiD) from Kaggle. The datasets used were relatively smaller and may not represent the global diversity of retinal disease presentations. The FedProx aggregation improved performance in non-IID scenarios; however, a comparison with other advanced aggregation techniques, such as MOON or FedNova, should be explored further.

Gulati *et al*., in 2024 [20], also proposed a collaborative and privacy-focused approach to medical diagnostics using a Federated Deep Learning (FDL) framework with a lightweight MobileNetV2 architecture and FedProx aggregation. The authors analyzed the effect of varying µ values in the FedProx algorithm, showing that higher µ values stabilize training and enhance performance metrics. The decentralized approach enables the utilization of distributed resources, thereby minimizing bandwidth and reducing central server load. Data augmentation techniques, such as flipping and cropping, enhance the robustness of the model against overfitting. The systematic evaluation of proximal term values ensures optimal trade-offs between accuracy and stability. The simulation of three clients might not capture the complexities of real-world deployments involving hundreds of institutions. Future directions include enhancing privacy with differential privacy and secure multi-party computation techniques.

Otoum *et al*. (2024) [109] introduced an FL framework for heart disease prediction using TabNet, differential privacy, and blockchain. The authors used the UCI and Comprehensive Heart Disease datasets. Their proposed model achieved an accuracy score of 82.2% with a balanced privacy-performance score of 1.594 at 50 training epochs. The blockchain-based smart contracts reinforced transparency and enhanced the security of data. However, the system exhibited inconsistent performance across datasets, particularly lower accuracy on the smaller UCI dataset, and incurred computational overhead due to blockchain integration. The work’s focus on tabular data may limit generalization to imaging-based clinical diagnostics. Future work should explore hybrid models, improve interpretability through explainable AI, and enhance privacy without sacrificing accuracy.

An analytical summary of the FL models used for detecting DR and DME is presented in Table **6**.

### 3.4 An Analytical Discussion on Disease Detection Models Trained through Federated Learning

This section examines the use of FL models for detecting diseases and addresses their drawbacks while providing the key findings.

Roth *et al*., in 2020 [98], implemented a distributed system using FL, specifically the FedAvg algorithm for global model updates, and selected participants using mini-batches of size 32. The global model underwent 300 training rounds over approximately 36 hours. The real-world dataset, sourced from seven medical organizations, consisted of 142,952 mammography images. This non-IID and heterogeneous dataset varied significantly in breast density classifications. The model's performance was evaluated using Cohen’s Linear Weighed Kappa score, both before and after applying federated models and further fine-tuning. However, the study lacks several key aspects, including other aggregation strategies, communication efficiency, and privacy algorithms, which have not been explored. Additionally, it does not address the challenges of class imbalance and data size heterogeneity. The metrics essential for the performance analysis and the model parameters used in implementation are also not clearly mentioned in the study. Implementing the model using a very large dataset requires significantly more computational power, and it takes a considerable amount of time to converge.

Zhang *et al*. (2021) [99] introduced an FL diffusion technique with two decision-making processes: client participation and client selection. The experiment involved two types of medical images: CT and X-ray images. The CT dataset consisted of 746 images, and the X-ray dataset comprised 2960 images. Three different models were used: GhostNet, ResNet50, and ResNet101, and their accuracies were compared across various dataset partitions. The authors concluded that this approach improved accuracy but required more training time compared to the standard federation model. The study does not specify the training details, including the learning rate and number of epochs. Additionally, performance metrics and model parameters have been omitted. The proposed work utilized deep neural networks with 101 layers, which are not only more time-consuming and less scalable but also overburden resources with increased computational requirements.

Yang *et al*. (2021) [100] developed an FL method for segmenting and classifying 3D chest images for COVID-19, utilizing semi-supervised learning to reduce annotation requirements and maintain data privacy. The approach, tested with data from three hospitals in Italy, China, and Japan, involved both labeled and unlabeled datasets. It showed improved performance with frequent aggregation rounds in an ablation study. A CNN-based model with augmented data was used for analysis. The study combined datasets from five public sources, including 28833 X-ray images. The proposed model achieved an accuracy comparable to traditional models (92.24%) but was about ten times faster. The article, however, does not take into consideration the variability in the annotation or labelling of images by different ophthalmologists. Additionally, data privacy over the network is not addressed during the model's implementation. The model parameters used in simulating the FL setup are not explicitly provided. The other aggregation strategies, such as FedProx, can also be used to further improve accuracy.

Khan *et al*., in 2021 [101], used FL for pneumonia detection from chest X-rays, focusing on privacy by not sharing data centrally. The study used a dataset of 5856 pneumonia images (excluding COVID-19 cases), which were split into 4684 training and 1172 testing images. A Canny filter was applied in pre-processing. Simulating two client hospitals and a server, the study compared four deep-learning models: CNN, ResNet50, VGG16, and AlexNet. The VGG16 model performed better than the other two in comparison based on the accuracy and other performance across metrics like precision, recall, F1-score, and sensitivity, making it the preferred model for this application. The study does not specify the aggregation method used in the experimental setup, and the number of communication rounds is quite low, which should be increased for the global model to converge and stabilize.

Balaji *et al*., in 2021 [102], evaluated an FL model for pneumonia classification using chest X-ray images (CXR), comparing it with a centralized model and examining three aggregation strategies: FedAvg, COMED, and GEOMED. COMED is a median-based strategy, and it resists model alterations by selecting the median value from sorted parametric values. GEOMED aims for a geometric median in model aggregation, seeking a global minimum. The study utilized the ImageNet-1K dataset, comprising 5856 images, which were split into training and test sets. The COMED and GEOMED outperformed FedAvg in terms of robustness. Four CNN-based DL models (AlexNet, ConvNet, ResNet18, and ResNet50) were used for training and evaluated on accuracy, precision, recall, and F1 score. There was a trade-off between communication efficiency and prediction accuracy. Moreover, the accuracy score needs to be improved. Additionally, the model suffers from slow convergence due to a small learning rate.

Kumar *et al*. (2021) [103] created an FL model for COVID-19 detection using CT scans, with a dataset (CC-19) comprising 34,006 images from 89 subjects across three hospitals. The approach involved normalizing data, using DL and SegCaps for image segmentation, and a Capsule Network for training. The federated model involved local training by clients and central weight aggregation. Although the model achieved a good accuracy score, the latency due to communication and using other communication-efficient techniques was not considered. The model parameters, such as epochs, communication rounds, clients, *etc*., were also not specified for a better comparison with state-of-the-art research. Furthermore, the approach is specific to the application and cannot be applied to other diseases.

Wu *et al*., in 2022 [104], developed the FedRare method for detecting rare diseases in imbalanced data situations, using an FL model to extract and classify latent features through contrastive supervised learning. Tested on the HAM10000 dataset, which includes data from two rare diseases, the method involved dividing the data into training, validation, and testing sets, with adjustments made for fair evaluation. The model employed contrastive learning for local training and FedAvg for server aggregation. FedRare's performance, as measured by balanced accuracy, surpassed that of other models, including FedAvg, FedProx, and MOON, demonstrating its effectiveness in rare disease identification. However, the developed technique needs to counter the imbalance in data, and both IID and non-IID scenarios should be considered for comparison. Furthermore, the accuracy achieved by the proposed method is not satisfactory and requires considerable improvement.

Zhang *et al*., in 2022 [105], introduced a heterogeneity-aware FL approach, named the SplitAvg method, to enhance federated model performance amidst data heterogeneity. Utilizing three varied datasets, 44,351 retinal images from the Kaggle Diabetic Retinopathy Competition, 14,236 paediatric radiographs from Kaggle's BoneAge set, and a manually collected dataset of 165 subjects from various institutions, the research compared SplitAvg with seven well-known FL methods. The focus was primarily on the ability to manage heterogeneous data, which was assessed primarily by classification accuracy. Despite this, device heterogeneity and behaviour heterogeneity were not considered. Moreover, security of the method needs to be implemented to prevent the risk of data leakage arising from shared feature maps of the cut layer in the architecture.

An FL aggregation model called FedFocus was proposed by Li *et al*. (2022) [106] to detect COVID-19 in chest X-ray (CXR) images. The authors used real-world data comprising 16689 chest X-ray images from three Chinese cities, and a ten-client simulation was performed. FedFocus, differing from the average aggregation of FedAvg, updated global parameters based on a weight loss-dynamic tuning parameter after each client training round. The ResNet-18 architecture was utilized for both global model training and local client training. Although the training accuracy of FedFocus was improved over that of FedAvg, a very large dataset was used, and the excessive number of communication rounds (500) and clients (10) contributed to the communication overhead and latency, requiring high computational power. Additionally, the performance with an IID scenario was not compared, and the results were obtained only under non-IID settings. The performance relies on the number of training samples, which can be a limitation in cases where data is scarce or highly imbalanced.

An approach for classifying breast cancer was proposed by Tan *et al*. in 2023 [107]. This framework utilizes transfer learning techniques to extract and enhance data features from mammography images, leveraging pre-trained models, such as MobileNet and ResNet50. The Federated Averaging (FedAvg) algorithm was used at the server to aggregate the updates from the clients. The Synthetic Minority Oversampling (SMOTE) technique was used to augment data in minority classes and address the issue of data imbalance. After testing numerous popular CNN models, MobileNet proved to be suitable for federated settings due to its lower computational requirements and comparable performance to larger models, such as ResNet. The achieved performance indicated that the FL framework can manage non-IID data, albeit with reduced performance. SMOTE and transfer learning help in managing non-IID data, but they are unable to fully bridge the gap compared to IID data. Future work could explore advanced aggregation schemes and domain adaptation methods to further enhance the performance of these systems. Future research could explore extending the methodology to cover other cancers and integrating real-time feedback systems for radiologists.

Tanim *et al*. (2024) [49] worked on the detection of Parkinson’s disease. Dirichlet-based data partitioning was introduced to address label skew and balance data distribution across clients. CKKS homomorphic encryption was employed to securely aggregate model updates, preserving privacy without compromising performance. An attention-based fusion model for dynamic weight assignment to local updates was implemented to ensure better integration into the global model. The authors utilized the PD-BioStampRC21 dataset, which features clinical and sensor data from patients with Parkinson’s disease and a control group, and compared federated learning algorithms (FedAvg, FedProx, and SCAFFOLD) under non-identically distributed (non-IID) settings. The FedProx aggregation scheme demonstrated moderate stability and accuracy improvement, while SCAFFOLD effectively corrected local update drifts but exhibited sensitivity to data skew. The proposed method achieved a global model accuracy of 64.40%, significantly outperforming the random partitioning approach, which achieved an accuracy of 57.77%. The dataset's small sample size of 17 participants per group limits statistical power and fails to capture the diversity of patient populations. The CKKS homomorphic encryption scheme, while effective in maintaining privacy, adds significant computational overhead and latency, which may hinder real-time applications. Despite the improvements made, the global model’s accuracy (64.40%) still reflects challenges in effectively managing non-IID data distribution. In the future, the dataset can be expanded to include a larger and more diverse participant pool to enhance model generalizability. Additionally, lightweight encryption methods or hybrid techniques should be developed to balance privacy preservation with computational efficiency.

Table **7** presents a summary of the disease detection models trained through federated learning, including methodology, model parameters, performance, and research gaps.

### 3.5 Open Issues and Research Challenges in Federated Learning

Federated Learning (FL) is a rapidly evolving field, and with its growth comes various open issues and research challenges. The following are some of the key areas where research and development are particularly active:

#### 3.5.1 Data Privacy and Security

Data privacy and security are central concerns in FL, as the primary goal of it is to learn from decentralized data without compromising the privacy of individual data sources. Two commonly used techniques for securing FL parameters over the communication medium are differential privacy and secure multiparty computation.

Differential Privacy (DP) is a framework for measuring and controlling the privacy risks involved in communicating information, such as model weights and parameters derived from sensitive data [110].

Secure Multi-Party Computation (SMPC) is a cryptographic technique that enables multiple collaborators to compute the same function with their own local inputs while maintaining the privacy of those inputs [57].

However, implementing differential privacy (DP) in federated learning involves a trade-off between privacy and model accuracy. This is because when noise is added to model updates to protect individual data, the efficiency of the model often degrades. Additionally, DP introduces computational overhead due to noise generation and privacy accounting, necessitating careful tuning of privacy parameters to strike a balance between utility and privacy.

Meanwhile, secure aggregation provides strong cryptographic guarantees by enabling the server to aggregate client updates without accessing individual data, preserving privacy without impacting model accuracy. However, this method incurs significant communication and computational costs due to the cryptographic operations and multiple communication rounds involved. The secure aggregation protocols also face challenges with client dropouts, which can complicate protocol completion and affect scalability.

Overall, differential privacy may reduce model performance but is more robust to client variability, while secure aggregation maintains accuracy but demands greater system complexity and resource consumption.

#### 3.5.2 Communication Efficiency

Communication efficiency is a critical aspect of an FL framework, given its foundational nature as a distributive and decentralized approach. In the framework, training of the central/global model occurs using multiple devices or servers, each holding local data and connected to a network [111, 112]. These participating devices send their local model updates, such as gradients, parameters, or weights, to the central server for aggregation, thereby contributing to global learning. This process is communication-intensive, and communication efficiency becomes pivotal when key models are large and the bandwidth is limited [113-115]. Therefore, improving communication efficiency is essential to make FL feasible and efficient in real-world scenarios. Communication efficiency can be achieved by two techniques: model compression and efficient update aggregation [73].

Model compression aims to reduce the size of the model or the updates that need to be communicated. Model compression can be achieved by parameter pruning and quantization, sparse updates, and knowledge distillation [116, 117].

Efficient update aggregation techniques are used to reduce the frequency or volume of data exchange without compromising the learning process. Some of the methods of achieving this are: federated averaging, decentralized aggregation, and asynchronous updates [118].

#### 3.5.3 Heterogeneity in Data and Systems

Federated frameworks often comprise devices with varying computational capabilities and datasets of different sizes and features. The heterogeneous nature of devices and data creates a challenging environment, making consistent model performance across the network a difficult task.

Thus, the heterogeneity in datasets and the systems used is a significant challenge in an FL setup. The heterogeneity in data is introduced by variability in data distribution, imbalanced data, improper quality of data (poorly labelled), and noise in data. On the other hand, heterogeneity in systems is due to the variability in computational resources used and the variation in network connectivity.

#### 3.5.4 Scalability

The number of devices in a federated network is crucial; as the number of devices increases, maintaining efficient and effective training becomes even more challenging. The research should focus on designing and developing scalable algorithms and architectures that can handle large-scale federated learning without any significant drop in performance [119]. In practicality, FL systems are often large networks, and ensuring that the learning process remains efficient and effective while dealing with the increased complexity and diversity of devices and data is a notably challenging task. The main challenges faced include a large computational load, communication overhead, and heterogeneity in both data and systems [120].

#### 3.5.5 Robustness and Fault Tolerance

One of the critical aspects of an FL system is to ensure that it is robust against failures and malicious attacks. To ensure robustness, strategies to detect and mitigate issues, such as model poisoning and dealing with unreliable or compromised devices in the network, need to be designed [58].

## CONCLUSION AND FUTURE DIRECTIONS

The systematic review provides a thorough and insightful illustration of the deep learning and federated learning techniques utilized to detect ocular diseases. The transformative impact of AI in healthcare diagnostics emphasizes the critical role of federated learning in enhancing data privacy, a paramount concern in today's digital age, especially in the healthcare domain. The application of AI in healthcare has significantly improved the performance of automated disease diagnostic systems. These advanced AI systems demonstrate a remarkable ability to understand and analyze complex medical images, contributing to the early and more accurate detection of diseases, which is crucial for effective treatment strategies. Furthermore, the review explores the evolution and application of federated learning in the healthcare domain. The emerging distributed technology is quite promising in addressing the significant challenges posed by traditional AI models. The decentralized framework helps protect sensitive patient data.

As the application of deep learning and federated learning in healthcare continues to evolve, the focus of future research should be to refine these technologies significantly. One of the primary focus areas is the amplification of data privacy and the preservation of data utility. This includes complex encryption approaches and innovative algorithms designed to extract insights from data while safeguarding individual data elements. Balancing data utility with privacy is of prime importance to ensure that patient data remains informative for diagnostics while being secure from unauthorized access. Apart from privacy, scalability is another critical factor.

Healthcare data is experiencing exponential growth, and scalable federated learning systems are crucial for accurate disease diagnosis. In the future, it is recommended that algorithms and architectures be developed to efficiently process large datasets across diverse settings. The vital concerns in an FL-based detection system are optimizing communication protocols, decreasing computational loads on individual nodes, and adapting to various device capacities and network capabilities. Improving both the robustness and accuracy of AI models for disease diagnosis remains an essential goal. The potential of these technologies to revolutionise healthcare is immense, promising enhanced healthcare outcomes and enriched patient experiences.

Furthermore, it is worth noting that differences in datasets, data distribution, and hardware make it challenging to generalize results or establish consistent evaluation standards. The communication efficiency, scalability, and advanced privacy techniques can be explored for deployment in real-world scenarios to achieve better generalizability of the federated deep learning model. The model’s robustness, fairness, and its integration into clinical settings are still open research challenges that need to be addressed in the future.

## Figures and Tables

**Fig. (1):**
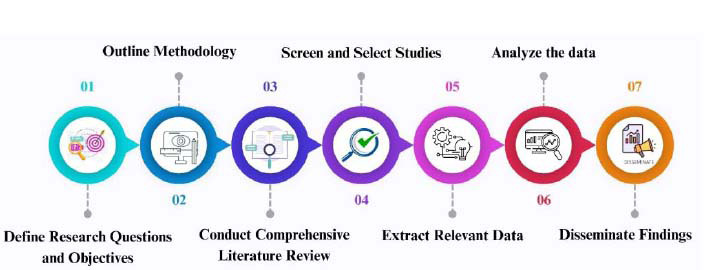
The research methodology of the systematic review.

**Fig. (2):**
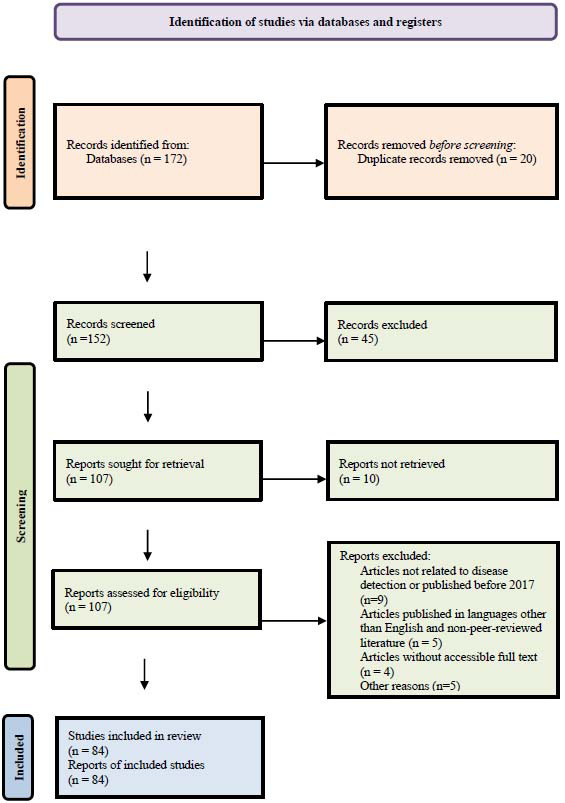
The PRISMA flow diagram for the systematic review.

**Fig. (3):**
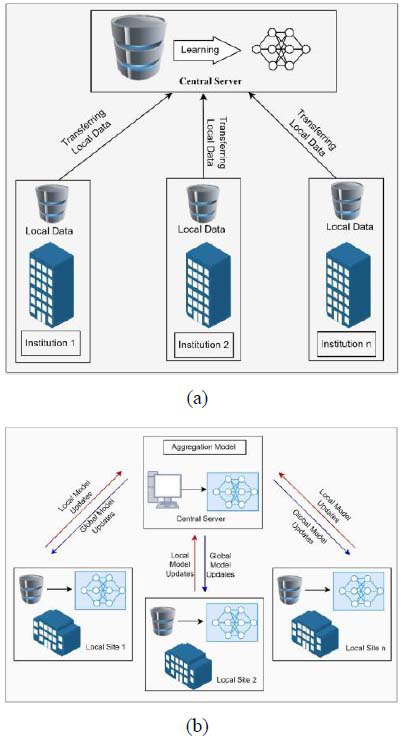
The architecture of a (**a**) traditional centralized deep learning model and (**b**) federated deep learning model.

**Fig. (4):**
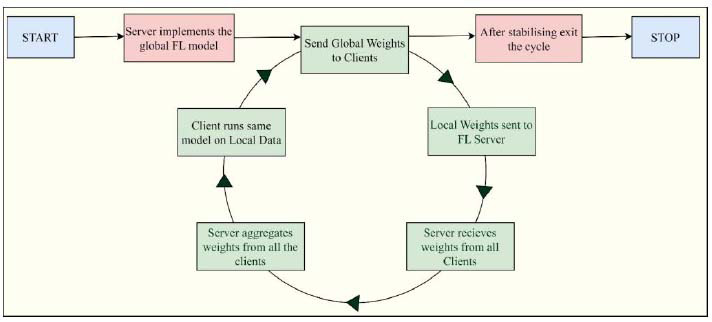
Illustrating the iterative behaviour of the federated learning approach.

**Fig. (5):**
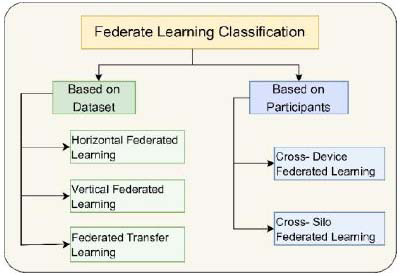
Classification of federated learning.

**Fig. (6):**
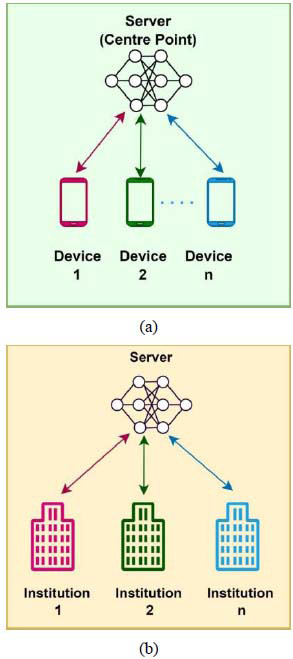
Diagrammatic representation of **(a)** Cross-Device and **(b)** Cross-Silo Federated Learning.

**Fig. (7):**
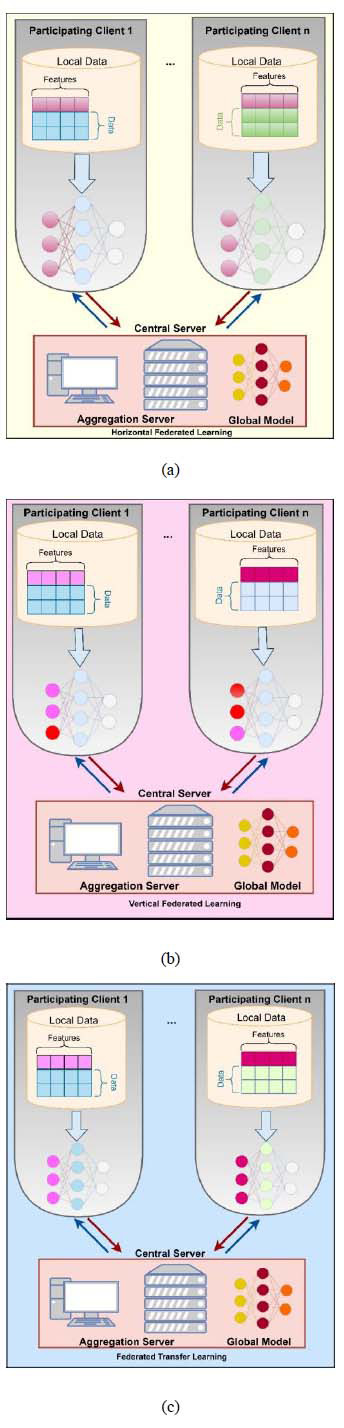
Characterization of Federated Learning: (**a**) Horizontal, (**b**) Vertical, (**c**) Federated Transfer.

**Table 1:** Comparative analysis of the different survey articles on federated learning.

**Reference**	**Focus Area**	**Review/ Survey Methodology**	**Limitations and Recommendations for Future Research**
A Review of Applications in FL [27]	Discussion on the applications of FL in mobile, industrial, and healthcare.	Detailed literature review	• Efficient communication strategies and server-side aggregation strategies have not been addressed. • Broad research directions need to be explored further.
A Systematic Review of FL Applications for Biomedical Data [28]	Evaluation of FL applications in medicine, focusing on oncology and radiology.	Systematic review of studies (PRISMA)	• Lacks discussion on parameter aggregation, global model update techniques, and communication-efficient strategies. • Improving FL methodologies in healthcare and addressing bias issues should be explored.
A Comprehensive Survey of Privacy-Preserving FL: A Taxonomy, Review, and Future Directions [29]	Identification of privacy leakage risks.	Categorizing privacy risks and PPFL methods using the 5W framework: Who, What, When, Where, Why.	• High computational needs are not addressed. • Ethical and fairness issues remain underexplored.
FL Review: Fundamentals, Enabling Technologies, and Future Applications [30]	In-depth overview of FL in AI, IoT, blockchain, and various markets.	Comprehensive literature review	• Focuses more on system design than practical implementation challenges. • Missing privacy-preserving mechanisms and communication methods for system convergence. • Future work should be directed toward recommendation engines, IoT, privacy, and public sector use.
FL in a Medical Context: A Systematic Literature Review [31]	A systematic review of FL in the healthcare sector with a focus on oncology and radiology.	Systematic literature review (PRISMA)	• Does not discuss the parameter aggregation and different techniques to update the global model. • Communication-efficient strategies are also not included. • In the future, enhancing data privacy and addressing bias in medical FL applications should be considered.
A Survey on FL Systems: Vision, Hype, and Reality for Data Privacy and Protection [32]	Developing FL systems; categorization based on various aspects; analyzing system components.	Comprehensive review and categorization of FL systems	• Highlights federated architectures and privacy mechanisms, but lacks specific applications of FL and discussion on various aggregation methods and communication efficiency. • Future research should focus on the development and infrastructure improvements of FL systems.
Survey on FL [33]	Explores FL's privacy aspects	Survey and analysis	• The evolution of FL, its characteristics, categories, and challenges have not been considered. • The works should focus on privacy enhancements.
Towards Efficient Communications in FL: A Contemporary Survey [34]	Evaluation of communication methods in FL; client heterogeneity; privacy concerns	Methodical evaluation of FL communication research	• Focused mainly on communication aspects, potentially overlooking other FL challenges like aggregation methods and secure communication methods. • Unaddressed background, like FL classifications. • In the future, enhancements in communication efficiency and resource allocation in FL.
The Current State and Challenges of Fairness in Federated Learning [35]	Addresses fairness issues in FL systems	An analytical and conceptual review	• Limited coverage of categorization, communication efficiency, practical applications, and implementations. • The research is too specific and focuses solely on fairness in FL.
FL for Generalisation, Robustness, Fairness: A Survey and Benchmark [36]	Foundational concepts, key challenges, and methodological developments	A systematic review	• Architectural classification of FL based on datasets and devices has not been discussed in detail. • Reliability on traditional benchmark datasets (CIFAR-10 and MNIST), which do not represent the real-world, complex, and non-IID data.
A Review of FL Methods in Heterogeneous Scenarios [37]	FL challenges in heterogeneous scenarios focusing on device, data, and model heterogeneity.	Review analyzing impacts on training efficiency and accuracy.	• Limited coverage on large-scale deployment challenges. • Further research is needed on optimizing communication and adaptive methods for diverse heterogeneity.
A Survey for FL Evaluations: Goals and Measures [38]	Evaluation goals in FL focusing on utility, efficiency, and privacy.	Comprehensive review	• Limited privacy and security attack simulations. • Lacks standardised evaluation for heterogeneous FL, limited privacy-security trade-off analysis, underexplores vertical FL, asynchronous participation, and practical deployment. • Advanced privacy methods have not been deeply analysed.
Decentralized FL: A Survey and Perspective [39]	Decentralized FL advantages and real-world applications.	Comprehensive survey	• Lacks frameworks for decentralised coordination, conflict resolution, and deadlock prevention. • Needs scalable techniques for handling extreme client heterogeneity.
FL with Non-IID Data: A Survey [40]	FL under non-IID data conditions: communication, convergence, bias, privacy, and security.	Comprehensive survey	• Does not address real-world scaling and device limitation challenges. • Privacy and security issues specific to non-IID data are not fully covered.

**Table 2:** Summary of machine learning and deep learning models in ocular disease detection.

**Reference**	**Focus**	**Methodology**	**Dataset**	**Key Performance Metrics**
Rekhi *et al*., 2017 [74].	DME Detection	Morphological features, SVM	MESSIDOR, DIARETDB1	Achieved 92.11% accuracy for severe DME (DIARETDB1), 90% accuracy for severe DME (MESSIDOR), and above 87% for moderate cases; robust 4-step method, including normalization and exudate segmentation.
Marin *et al*., 2018 [75]	DME Risk Detection	Supervised learning	1058 fundus images	Good sensitivity scores 90%, moderate specificity scores 70%; potential for improved performance.
Qummar *et al*., 2019 [76].	DR Staging	Deep CNN, Ensemble of 5 Models	35126 images from Kaggle	High accuracy in multi-class DR classification; use of an ensemble for improved prediction.
Zeng *et al*., 2019 [77]	RDR Detection	Siamese-like CNN	35126 fundus photos from Kaggle	Kappa score of 0.829; effective in detecting various DR severity stages.
Aamir *et al*., 2020 [78]	Glaucoma Detection	ML-DCNN	Public and private hospitals	Good sensitivity, specificity, and accuracy; effective multi-stage classification of glaucoma.
Selvathi *et al*., 2019 [79]	DED Detection Using Thermal Images	SVM	Thermal images from IGCAR; 283 in number	Fair accuracy score of 86.22%; effective for binary classification of healthy/diseased eyes.
Pan *et al*., 2020 [80]	Retinal Lesions Classification	Deep CNNs	4067 images from Zhejiang University	Identification of 4 lesion types; DenseNet most effective among the 3 CNN models.
Jiang *et al*., 2020 [81]	Segmenting the cup and the optic disc from the images	Joint RCNN	SCES, ORIGA datasets	Good AUC scores; improved joint segmentation performance.
Torre *et al*., 2020 [82]	DR Disease Grading	Interpretable DL classifier	85,650 images from EyePACS	Fair accuracy (85.7%); innovative score propagation model for pixel-wise classification.
Sarki *et al*., 2020 [83]	Mild/Multi-class DED Detection	Pre-trained CNNs	MESSIDOR, Drishti-GS, GitHub datasets	Good accuracy score; adhering to the BDA standards.
Karimi *et al*., 2024 [84]	Integration of DL models with EHR to improve early glaucoma detection	Used Random Forest, Gradient Boosting, and Sequential models	EHR data from 1,652 participants (826 glaucoma patients, 826 controls)	Random Forest achieved the best performance with 67.5% accuracy and a ROC AUC of 0.67.
Gao *et al*., 2024 [85]	Fundus photograph-based cataract evaluation	DCEN using ResNet50 for classification and severity grading	1,340 color fundus photographs	Achieved 97.62% accuracy, 94.01% F1-Score, and 0.8618 Kappa score for cataract type classification.
Elkholy *et al*., 2024 [86]	Classification of OCT images into 4 categories: normal retina, DME, CNM, and AMD	Image preprocessing, Transfer learning-VGG16 model	35,468 OCT images from the public dataset on Kaggle	Achieved 97% classification accuracy across 4 categories (Normal, DME, CNM, AMD); used image enhancement and fine-tuning; noted dataset imbalance challenges for real-world applications.
Deepak *et al*., 2024 [87]	Classifying ocular diseases- cataract and glaucoma	Transfer learning- EfficientNetB0, SqueezeNet, DarkNet-53; Optimized batch size and optimizers	10,000 fundus images from the Ocular Disease Intelligent Recognition (ODIR) dataset from Kaggle	DarkNet-53 achieved the highest accuracy (99.4%) with a batch size of 6 and the SGDM optimizer. The complexity of the DarkNet53 model may cause issues in model deployment.

**Table 3:** Overview of the federated learning models in ocular disease detection.

**Reference**	**Focus Area**	**Methodology**	**Dataset**	**Key Findings**
Matta *et al*., 2023 [88]	Diabetic Retinopathy Detection	EfficientNet-B5 (pre-trained) Weighted/ Unweighted Averaging	OPHDIAT, 697275 Images	Comparable AUC to centralized models; privacy-preserving
Tang *et al* 2023., [89]	Multi-Disease Ocular Recognition	Gaussian Randomization and pre-trained ResNet50	OIAODIR, 10000 Images	Enhanced cross-domain classification; data confidentiality
Nasajpour *et al*., 2022 [90]	Diabetic Retinopathy Detection	FL with FedAvg and FedProx aggregation with AlexNet DL model	Multiple Public Domains, ~3000 Images	Accuracy comparable to centralized models; improved data security
Mohan *et al*., 2023 [91]	Diabetic Retinopathy Grading	Novel CNN Algorithm, FedAvg	MESSIDOR-2, IDRiD, Kaggle, 5000 Images	High accuracy, specificity, precision, F1 score; effective for diverse data.
Lo *et al*., 2021 [92]	Diabetic Retinopathy Classification	VGG19 with ImageNet Weights	Various Medical Institutions, 700 Images	Similar performance to centralized models, while maintaining data privacy
Hossain *et al*., 2023 [93]	Diabetic Retinopathy Prediction	AlexNet, ResNet50, SqueezeNet1.1, VGG16	35,120 Images	High accuracy; reduced communication overhead; balanced data privacy and accuracy
Khan *et al*., 2023 [94]	Cataract Detection Using Federated Learning (CD-FL)	Privacy-preserving FL framework using VGG16 as the backbone; training on clients and server	ODIR dataset, with 6392 images of normal eyes and cataract	Achieved a 95.28% accuracy score
Bhulakshmi *et al*., 2024 [95]	Diabetic Retinopathy Detection and Classification	FL framework with FedAvg using five CNN models ResNet50, DenseNet201, AlexNet, EfficientNetB7, and VGGNet19	IDRiD dataset with 516 images and STARE dataset with 400 images for generalization	ResNet50 achieved highest accuracy of 94.66%
Gulati *et al*., 2024 [23]	Federated Learning Framework for Detecting Retinal Diseases	Backbone model: MobileNetV2 with ImageNet weights. FedAvg and FedProx aggregation.	Pool of 2 sources: Eye Fundus and OCT images of (Translational Visual Health Laboratory) and the Indian Diabetic Retinopathy Image Dataset (Kaggle)	FedAvg achieved 98.69% accuracy with IID data and 87.09% with non-IID, while FedProx reached 98.03% with IID and 98.25% with non-IID data.
Gulati *et al*., 2024 [20]	Detection of Diabetic Eye Diseases (DEDs)	FL framework using MobileNetV2 and FedProx aggregation strategy	Combined dataset of 2,700 images from OCT and Eye Fundus Dataset, IDRiD, and ODIR datasets	Achieved 98.7% accuracy; highlighted stability with FedProx for heterogeneous data.
Jiang *et al*., 2025 [96]	Fundus Disease Diagnosis	DataWeightedFed: random k-client selection + weighted FedAvg aggregation	Subset of ODIR with 3 classes and 3,402 images	Closed central-vs-FL accuracy gap to 1.85%; efficient training (5 FL rounds ≃ 5 CL epochs); limited by small client count, narrow disease range, no privacy/communication metrics.
Gulati *et al*., 2025 [97]	DR and DME Detection	Horizontal FL with MobileNetV2 backbone; FedAvg aggregation	Kaggle composite with 838 images consisting of 278 DME, 280 DR, and 280 normal images	96% average accuracy; 100% class-wise recall for DR and DME; constrained by a small dataset and the use of plain FedAvg without stronger privacy-focused aggregation like FedProx.

**Table 4:** Summary of literature review on federated learning for disease detection.

**Reference**	**Disease**	**Methodology**	**Dataset**	**Key Findings**
Roth *et al*., 2020 [98]	Breast density classification	FedAvg, TensorFlow, NVIDIA Clara Train SDK	142,952 Mammography images	A 6.3% accuracy improvement in distributed training and a 45.8% improvement in generalizability
Zhang *et al*., 2021 [99]	COVID-19 detection	Dynamic-Fusion, GhostNet, ResNet50, ResNet101	3706 CT and X-ray images	Better accuracy but longer training time than the default federation model
Yang *et al*., 2021 [100]	COVID segmentation in chest CT	Federated semi-supervised learning	1704 Chest CT scans from China, Italy, and Japan	Improved segmentation in multinational data; better self-supervised model performance
Khan *et al*., 2021 [101]	Pneumonia detection	Federated Learning, CNN models	5856 Chest X-Ray Images	The VGG16 model was most effective, achieving 91% accuracy
Balaji *et al*., 2021 [102]	Pneumonia image classification	FedAvg, COMED, GEOMED, SMPC	5856 Chest X-Ray Images	COMED and GEOMED are more resilient to outliers; better accuracy compared to FedAvg
Kumar *et al*., 2021 [103]	COVID-19 detection using CT imaging	Blockchain-Federated-Learning, Capsule Network	34,006 CT Images	High accuracy (98.68%) and better performance than traditional models
Wu *et al*., 2022 [104]	Rare disease classification	FedRare, Contrastive Learning	11,720 Images from HAM10000	9.6% increase in balanced accuracy; effective for class imbalances
Zhang *et al*., 2022 [105]	Diabetic retinopathy, bone age prediction, brain tumor segmentation	SplitAvg: an FL approach for data heterogeneity	Retinal images- 44,351 from Kaggle; BoneAge images-14,236 from Kaggle; BraTS: Brain tumor images- 285 subjects collected from multiple sources	96.2% accuracy for diabetic retinopathy detection
Li *et al*., 2022 [106]	COVID-19 detection	Federated Learning with CNN – FedFocus, ResNet-18 backbone	COVIDX8 dataset with 16,689 chest X-ray images	Average test accuracy of 92.09%
Tan *et al*., 2023 [107]	Breast cancer detection	FeAvg-CNN + MobileNet, Transfer Learning, SMOTE	DDSM and CBIS-DDSM mammography datasets	Achieved nearly 98% accuracy; improved class balance and privacy in federated settings
Tanim *et al*., 2024 [49]	Parkinson’s disease	CKKS Homomorphic Encryption, Attention-Based Fusion, Dirichlet Data Partitioning	PD-BioStampRC21 dataset	Enhanced accuracy and robustness; effectively addressed non-IID data issues and ensured privacy
Mitrovska *et al*., 2024 [108]	Alzheimer's disease detection	Federated Averaging, Secure Aggregation, 3D CNN	ADNI: 618 subjects	Secure FL achieved accuracy close to that of centralized training; however, performance decreased with highly non-IID client data, and non-IID strategies were not explored
Otoum *et al*., 2024 [109]	Heart disease prediction	Federated Averaging with TabNet, Blockchain	Comprehensive heart disease and UCI heart disease	Accuracy about 82% after 50 rounds; blockchain adds computing overhead; smaller datasets cause overfitting

**Table 5:** Analytical summary of ML and DL techniques used for detecting DR and DME.

**Reference**	**Key Points**	**Dataset Description**	**Shortcomings**	**Future Scope**
Rekhi *et al*., 2017 [74]	Detects DME using morphological features, a 4-step methodology, and small databases.	Publicly available DIARETDB1 dataset with 89 digital-coloured images and MESSIDOR with 1,200 high-resolution fundus images.	Small database, potential overfitting, and limited assessment in a clinical setting.	Expand to diverse datasets, assess clinical performance, and improve algorithm efficiency.
Marin *et al*., 2018 [75]	Identifies DME risk using fundus photos, tested on 1058 images, with supervised learning, specificity ~70%, sensitivity ~90%.	MESSIDOR database consisting of 1,058 macula-centred retinographies from 529 diabetic patients.	Performance can be improved, but it lacks computational efficiency analysis and has limited generalization.	Enhance system performance with more training compared to existing methods and explore scalability.
Selvathi *et al*., 2019 [79]	Detects DEDs using thermographic images, SVM algorithm, a dataset of 283 images, with accuracy 86.22%, specificity 79.17%, and sensitivity 94.07%.	Privately collected dataset of 283 thermal images of the eye.	Limited dataset, uncommon imaging method, not integrated into clinical workflows.	Improvement in robustness and generalizability, as well as integration into clinical workflows.
Qummar *et al*., 2019 [76]	Classifies DR into five stages using a Deep CNN, trained on 35,126 images, 80.8% accuracy, and an ensemble approach.	Publicly available Kaggle Diabetic Retinopathy Detection dataset that contains a total of 35,126 colour fundus images.	Dataset imbalance, computational complexity, and limited generalizability.	Improving accuracy, addressing dataset bias, and simplifying for practical deployment.
Zeng *et al*., 2019 [77]	Developed a Siamese network-based architecture using deep CNNs for detecting RDR.	Kaggle Diabetic Retinopathy Detection dataset, provided by EyePACS, consisting of 35,126 high-resolution color fundus images.	High computational demands and potential risk of overfitting due to model complexity.	Enhance efficiency and validate clinically, and apply regularization to reduce overfitting.
Aamir *et al*., 2020 [78]	Classifies glaucoma using ML-DCNN, Dataset: 4,067 FFA images (Zhejiang University). DenseNet outperformed VGG16 and ResNet50.	1338 retinal fundus images collected from various private and public hospitals.	Computational challenges and potential overfitting; an imbalanced dataset and limited validation.	Optimize efficiency and address overfitting, expand validation on diverse datasets, improve model accuracy for lesion detection.
Jiang *et al*., 2020 [81]	Detects glaucoma using Joint RCNN, datasets from SCES and ORIGA, and improved AUC values.	The ORIGA dataset contains 650 retinal fundus images, and the Singapore Chinese Eye Study (SCES) dataset consists of 1,676 images.	Primarily designed for glaucoma, lacks detailed performance metrics.	Expand the application to other eye diseases, and need detailed validation.
Torre *et al*., 2020 [82]	Classifies DR severity using DL, the dataset from EyePACS, with an accuracy of 85.7%, and potential overfitting.	EyePACS dataset of the diabetic retinopathy detection with 35,126 retinal fundus images.	Large datasets may lead to overfitting and not reaching benchmark models.	Improve accuracy, facilitate benchmark comparisons, and mitigate overfitting.
Sarki *et al*., 2020 [83]	Timely diagnosis of DEDS by using transfer learning models (VGG16 and Inceptionv3) pre-trained on ImageNet, which was fine-tuned for datasets like MESSIDOR and DRISHTI-GS, with an accuracy of 88.3% (VGG16) and 81% (Inception V3).	Five different datasets available publicly. MESSIDOR dataset consisting of 1200 images, MESSIDOR-2 dataset consisting of 1748 images, DRISHTI-GS containing 101 retinal images, and cataract dataset acquired from Retina Dataset GitHub consisting of 100 cataract images.	The approach struggles with early-stage lesion detection and relies on small, open-source datasets that limit generalizability. Dataset imbalance needs ensemble models, and augmentation is required.	Integrating lesion segmentation techniques for better early-stage detection. Use larger and diverse datasets to develop domain-specific enhancements to improve classification accuracy. Use ensemble models to improve accuracy and counteract dataset imbalance.
Gao *et al*., 2024 [85]	Developed a DCEN for simultaneous classification and severity grading by combining features and achieved 97.62% accuracy for classification and 97.03% for severity.	The dataset used was from the Beijing Eye Study 2011, consisting of 1,340 retinal fundus images.	An imbalanced dataset (with few PSC cases), limited device variability, and a lack of demographic details introduce bias.	Use balanced and diverse datasets, including images from various devices, and incorporate demographic details.
Elkholy *et al*., 2024 [86]	Focus on detecting ophthalmic diseases like DME, CNM, and AMD using models like VGG16, achieving a classification accuracy of 97%.	A publicly available dataset from Kaggle, which consists of 35468 Optical Coherence Tomography (OCT) images of retinal scans.	The dataset is balanced, which is impractical and might lead to overfitting. The model relies on high-quality images, while real-world images from diverse sources may reduce performance.	Enhance the interpretation of OCT images by integrating advanced segmentation techniques for more accurate lesion analysis.
Deepak *et al*., 2024 [87]	Optimized CNNs (SqueezeNet, DarkNet53, EfficientNetB0) were used to detect cataracts and glaucoma, achieving 99.4% accuracy with DarkNet53 on the ODIR dataset.	The ODIR (Ocular Disease Intelligent Recognition) Dataset, available on Kaggle, consists of 5,000 color fundus images.	Limited disease categories in the ODIR dataset and reliance on high-resolution images reduce generalizability.	Add noise reduction and color correction to enhance robustness.
Jiang *et al*., 2025	Proposed a federated learning architecture using VGG-19, wFedAvg aggregation, and k-client selection, achieving 84.88% accuracy with only 1.85% drop from centralized learning.	Subset of the ODIR dataset (normal, glaucoma, cataract); 3402 fundus images, split across 4 clients.	Limited number of clients, balanced dataset distribution, reliance on high-quality pre-processed images, and limited disease categories.	Scale to more clients, test with diverse and non-IID data, expand to additional disease categories, and assess robustness with real-world image variability.
Gulati *et al*., 2025 [97]	FL framework using MobileNetV2 and FedAvg to detect diabetic retinopathy and macular edema. Achieved 96% accuracy and 100% class-wise accuracy (DR, DME) with 2–3 clients.	838 fundus images from two Kaggle datasets (Indian Diabetic Retinopathy and APTOS 2019), balanced across three classes: DME, DR, and normal.	Small, balanced dataset; limited generalizability; performance declines slightly with 4 clients.	Test on larger, non-IID datasets; explore differential privacy; enhance robustness across more client settings.

**Table 6:** An analytical summary of FL techniques used for detecting DR and DME.

**Article**	**Key Points**	**Dataset Description**	**Shortcomings**	**Future Scope**
Matta *et al*., 2023 [88]	Utilized FL for multi-center DR detection. Enhanced data privacy while enabling robust training across diverse datasets.	Retinal fundus images collected from various screening centers (the OPHDIAT screening network), 97275 fundus photographs, non-IID dataset.	Limited evaluation of dataset diversity. Potential challenges in communication efficiency across centers.	Expand to larger, more diverse datasets. Optimize communication protocols to improve scalability.
Tang *et al*., 2023 [89]	Combined FL with domain adaptation. Improved recognition for multiple ocular diseases. Strong focus on data privacy.	The OIA-ODIR dataset was used with 10,000 fundus images. The dataset is non-IID.	The complexity of integrating domain adaptation. Performance gaps on highly varied datasets.	Investigate advanced domain adaptation techniques. Apply the approach to a wider range of ocular diseases and datasets.
Nasajpour *et al*., 2022 [90]	Introduced federated transfer learning for DR detection. Utilized CNNs for improved accuracy.	Five publicly available diabetic retinopathy (DR) datasets: EyePACS with 1194 images, MESSIDOR with 578 images, IDRID with 312 images, APTOS with 703 images, and the University of Auckland (UoA) with 141 images. The datasets are non-IID.	The high computational cost of CNN models. Limited assessment of inter-device variability.	Explore lightweight models for improved efficiency. Evaluate model robustness across different hardware and imaging conditions.
Mohan *et al*., 2023 [91]	Focused on FL for DR grading. Provided a framework for privacy-preserving collaborative training.	The non-IID pooled dataset was sourced from MESSIDOR-2, IDRiD, Kaggle, and a local database from Silchar Medical College and Hospital, totalling 5,000 fundus images.	Relatively narrow application limited to grading tasks. Lack of validation on real-world, large-scale datasets.	Extend to additional DR tasks (*e.g.*, early detection). Integrate with real-world fundus imaging pipelines for validation.
Lo *et al*., 2021 [92]	Applied FL to OCT data for microvasculature segmentation and DR classification. Addressed privacy concerns in sensitive data.	Non-IID data from four OCT instruments and institutions- Simon Fraser University (SFU) and Oregon Health & Science University (OHSU), with 153 OCTA images for microvasculature segmentation and 700 eyes (OCTA and OCT) for RDR classification.	Challenges in segmenting fine microvasculature structures. Dependency on high-quality OCT images.	Investigate preprocessing methods to improve segmentation accuracy. Broaden the application to include additional ocular biomarkers.
Hossain *et al*., 2023 [93]	Proposed a differentially private FL framework. Enhanced privacy through differential privacy mechanisms.	The non-IID: Kaggle Diabetic Retinopathy Detection dataset was used with 35,120 retinal images.	Trade-offs between privacy and model accuracy. Limited testing on diverse, real-world data.	Explore advanced privacy-preserving mechanisms for better accuracy. Conduct studies with real-world data to validate framework robustness.
Khan *et al*., 2023 [94]	FL enables privacy-preserving decentralized training; VGG16 achieves high accuracy (95.28%) for cataract detection; superior performance compared to traditional models; ensures patient data confidentiality.	The Ocular Disease Intelligent Recognition (ODIR) database was utilized with 10,000 images.	Computational demands of VGG16 limit resource-constrained environments; communication overhead in FL; reliance on dataset quality; lack of raw data access hinders preprocessing.	Develop lightweight architectures; optimize communication protocols; expand datasets and validate with additional diseases; introduce FL-compatible data cleaning methods and statistical validation.
Bhulakshmi *et al*., 2024 [95]	The authors used FL for DR stage classification with ResNet50, achieving 94.66% accuracy on the IDRiD dataset and ensuring privacy by sharing model parameters.	Indian Diabetic Retinopathy Image Dataset (IDRiD) with 516 retinal fundus images.	Small, imbalanced dataset and reliance on FedAvg assuming uniform class distribution.	Pool data to form larger datasets, adopt advanced aggregation methods like FedProx, and integrate privacy techniques, such as differential privacy.
Gulati *et al*., 2024 [23]	Researchers addressed retinal disease detection (DR and DME) using federated learning with MobileNetV2 architecture and FedAvg/FedProx for data privacy and collaborative training.	A combination of two publicly available datasets: Translational Visual Health Laboratory Dataset with 1548 eye fundus images, and the Indian Diabetic Retinopathy Image Dataset (IDRiD) with 1548 images. The dataset is non-IID.	Small datasets (Translational Visual Health Laboratory and IDRiD) limit global diversity representation and lack comparison with advanced techniques like MOON or FedNova.	Expand the dataset to represent global diversity and explore advanced aggregation techniques (*e.g.*, MOON, FedNova).
Gulati *et al*., 2024 [20]	Privacy-focused FDL framework for detecting DR, DME, and glaucoma using MobileNetV2 and FedProx, analysing µ values for stability, leveraging decentralised resources, and using data augmentation for robustness.	A combination of four sources of fundus images: OCT and Eye Fundus Dataset, Indian Diabetic Retinopathy Image Dataset (IDRiD), Ocular Disease Intelligent Recognition (ODIR), and Robust Vessel Segmentation in Fundus Images.	Limited client simulation may not reflect real-world complexity.	Enhance privacy with differential privacy and secure multi-party computation.
Otoum *et al*., 2024 [109]	Combined TabNet, FL, differential privacy, and blockchain for heart disease prediction; achieved 82.2% accuracy and a balanced metric score of 1.594.	Comprehensive Heart Disease Dataset (1190 samples) and UCI Heart Disease Dataset (14 attributes); tabular data format.	Inconsistent performance across datasets, computational overhead from blockchain, and limited to tabular data.	Explore hybrid models, enhance interpretability through explainable AI, and maintain privacy without compromising accuracy.

**Table 7:** Summarisation of the disease detection models trained through federated learning.

**Reference**	**Dataset**	**Performance**	**Model Parameters**	**Research Gaps**
Federated Learning for Breast Density Classification [98]	142,952 Mammography images from 7 medical organizations.	A 6.3% improvement in performance and a 45.8% improvement in generalizability.	BS-32, rounds- 300, LR-0.0001, Optimizer- Adam, Aggregation- FedAvg.	• Other aggregation strategies, communication efficiency, and privacy algorithms are not explored. • Class imbalance and data size heterogeneity are not fully addressed. • Performance metrics and essential model parameters are missing. • For very large datasets, more computational power is required, and model convergence can take considerable time.
Dynamic-Fusion-Based Federated Learning for COVID-19 Detection [99]	Total of 3706 images (746 CT, 2960 X-Ray).	Better accuracy but more training time.	Backbone models- GhostNet, ResNet50, and ResNet101 models, Aggregation- FedAvg.	• Training details, such as the learning rate or epochs, are not specified. • Performance metrics and model parameters are not specified. • Deep Networks with 101 layers are more time-consuming and less scalable.
Federated Semi-Supervised Learning for COVID Region Segmentation [100].	1704 Chest CT scans from China, Italy, and Japan.	Federated model 92.26%, communication-efficient model 92.24%.	Backbone model- CNN, Batch Size-4, LR-0.0001, Aggregation- FedAvg.	• Other aggregation strategies are unexplored. • Annotation variability is not addressed. • Privacy of data over the communication medium is not handled. • Model parameters are missing.
A Federated Learning Approach to Pneumonia Detection [101].	5856 Chest X-ray images.	The VGG16 model achieved an accuracy of 0.91.	Backbone models: CNN, ResNet50, VGG16, and AlexNet, Optimizer- Adam, LR- 0.000001, Epochs-50, Rounds-1.	• The aggregation method is not specified. • Very few communication rounds (only 1). • Small dataset.
Investigating Federated Learning Strategies for Pneumonia Image Classification [102].	ImageNet-1K dataset with 5856 chest X-ray images.	COMED and GEOMED achieve better accuracy than FedAvg.	Backbone models-AlexNet, ConvNet, ResNet18, ResNet50, Optimizer- Adam, LR-0.000001, Aggregation- FedAvg, COMED, GEOMED.	• There is a trade-off between communication efficiency and prediction accuracy. Accuracy needs to be improved. • Slow convergence due to a small learning rate.
Blockchain-Federated-Learning for COVID-19 Detection Using CT imaging [103].	34,006 CT scan images from 89 subjects.	Accuracy of 98.68%, precision of 0.830, and sensitivity of 0.987.	Backbone model- SegCaps and Capsule Network for image segmentation and training. Optimizer- Adam, LR- 0.00001.	• Some model parameters are missing, including epochs, communication rounds, and clients. • Communication efficiency needs to be explored.
FedRare: Federated Learning for Rare Disease Classification [104].	HAM10000 dataset with 11,720 images of seven diseases.	FedRare achieves the highest average balanced accuracy at 79.7%.	Backbone model-EfficientNetB0, LR- 0.0005, BS- 64, Optimizer- Adam, Rounds- 70, Aggregation- FedAvg, FedProx, MOON, FedProc, FedIRM.	• Class imbalance is not countered. • Accuracy needs to be improved.
SplitAVG: Heterogeneity-Aware Federated Deep Learning [105].	Multiple datasets, including 44,351 retinal images and 14,236 paediatric radiographs.	SplitAvg shows 96.2% accuracy.	Backbone model- CNN, BS-32, LR-0.001, Optimizer-SGD, Aggregation- SplitAvg, FedAvg, FedSGD, CWT, and SplitNN.	• Only statistical data heterogeneity is considered; device heterogeneity and behaviour heterogeneity are not considered. • There is a risk of reconstructing raw images from shared feature maps of the cut layer; security should be implemented.
Integrated CNN and Federated Learning for COVID-19 Detection [106].	COVIDX8 dataset with 16,689 chest X-ray images.	FedFocus achieves 96.34% accuracy in COVID-19 detection.	Backbone models- ResNet-18, Clients-10, Rounds- 500, Epochs-5, LR-0.01, BS-128, Aggregation- FedAvg, FedFocus.	• Handling imbalanced datasets remains a challenge. • Performance relies on the number of training samples, which can be a limitation in cases where data is scarce or highly imbalanced.
A Transfer Learning Approach to Breast Cancer Classification in a FL Framework [107]	The study used the Digital Database for Screening Mammography (DDSM) with 2,620 scanned mammographic images.	The FL framework handles non-IID data but with reduced performance; SMOTE and transfer learning partially address this gap.	Backbone models- MobileNet, ResNet50, DenseNet121, Xception, Clients-4, Rounds-60, Epochs-3, LR-0.001, BS-128, Aggregation- FedAvg.	• Advanced aggregation schemes and domain adaptation methods need to be explored. • Extending the work to include other cancers and integrating real-time feedback systems for radiologists.
Secure FL for Parkinson’s Disease [49]	PD-BioStampRC21 (clinical and sensor data from Parkinson’s patients and controls).	Achieved 64.40% global model accuracy, FedProx showed moderate stability, and SCAFFOLD corrected local drifts but was sensitive to data skew.	Attention-based fusion and CKKS homomorphic encryption were used with Dirichlet-based partitioning; Clients- 2; Rounds-25; BS-128; Aggregation- FedAvg, FedProx, SCAFFOLD.	• A small dataset (17 participants per group) limits statistical power and diversity. • CKKS encryption adds computational overhead. • Challenges remain in managing non-IID data. • Future work includes expanding the dataset and developing lightweight encryption methods.

## Data Availability

All data generated or analyzed during this study are included in this published article.

## LIST OF ABBREVIATION

DL: Deep Learning
FL: Federated Learning
AI: Artificial Intelligence
ML: Machine Learning
PRISMA: Preferred Reporting Items for Systematic Reviews and Meta-Analyses
PPFL: Privacy-Preserving Federated Learning
FHE: Fully Homomorphic Encryption
SVM: Support Vector Machine
DR: Diabetic Retinopathy
RDR: Referable Diabetic Retinopathy
GLCM: Gray Level Co-occurrence Matrix
IGCAR: Indira Gandhi Centre for Atomic Research
NP: Non-Perfusion regions
DPN: Disc Proposal Network
CPN: Cup Proposal Network
SCES: Singapore Chinese Eye Study
ORIGA: Online Retinal Fundus Image Database for Glaucoma Analysis
EHR: Electronic Health Records
DCEN: Deep Cataract Evaluation Network
DME: Diabetic Macular Edema
CNM: Choroidal Neovascular Membranes
AMD: Age-related Macular Degeneration
AUC: Area Under the Curve
ODIR: Ocular Disease Intelligent Recognition
STARE: Structured Analysis of the Retina
FDL: Federated Deep Learning
CXR: Chest X-Ray
SMPC: Secure Multi-Party Computation
SMOTE: Synthetic Minority Oversampling Technique
DDSM: Digital Database for Screening Mammography
ADNI: Alzheimer's Disease Neuroimaging Initiative
FFA: Fluorescein Fundus Angiography
DEDs: Diabetic Eye Diseases
IOP: Intraocular Pressure
BMI: Body Mass Index
PSC: Posterior Subcapsular Cataract
DP: Differential Privacy
MESSIDOR: Methods to Evaluate Sensitivity to Diabetic Retinopathy
COMED: Coordinate-wise Median
GEOMED: Geometric Median

## STANDARDS OF REPORTING

PRISMA guidelines were followed.

## SUPPLEMENTARY MATERIAL

PRISMA checklist is available as supplementary material on the publisher’s website along with the published article.
